# A new computational framework for simulating airway resistance, fraction of exhaled nitric oxide, and diffusing capacity for nitric oxide

**DOI:** 10.1371/journal.pone.0311667

**Published:** 2025-01-30

**Authors:** Benoit Haut, Cyril Karamaoun, Clément Rigaut

**Affiliations:** Transfers, Interfaces and Processes, Université libre de Bruxelles, Brussels, Belgium; Vanderbilt University Medical Center, UNITED STATES OF AMERICA

## Abstract

In this paper, we present a new computational framework for the simulation of airway resistance, the fraction of exhaled nitric oxide, and the diffusion capacity for nitric oxide in healthy and unhealthy lungs. Our approach is firstly based on a realistic representation of the geometry of healthy lungs as a function of body mass, which compares well with data from the literature, particularly in terms of lung volume and alveolar surface area. The original way in which this geometry is created, including an individual definition of the airways in the first seven generations of the lungs, makes it possible to consider the heterogeneous nature of the lungs in terms of perfusion and ventilation. In addition, a geometry can be easily modified to simulate various abnormalities, local or global (constriction, inflammation, perfusion defect). The natural variability of the lungs at constant body mass is also considered. The computational framework includes the possibility to simulate, on a given (possibly modified) geometry, a test to measure the flow resistance of the lungs (including its component due to the not fully developed flow in the first generations of lungs), a test to measure the concentration of nitric oxide in the exhaled air, and a test to measure the diffusion capacity for nitric oxide. This is implemented in the framework by solving different transport equations (momentum and convection/diffusion) describing these tests. Through numerous simulations, we demonstrate the ability of our model to reproduce results from the literature, both for healthy lungs and lungs of patients with asthma or chronic obstructive pulmonary disease. Such a computational framework, through the possibilities of numerous and rapid tests that it allows, sheds new light on experimental data by providing information on the phenomena that take place in the distal generations of the lungs, which are difficult to access with imaging.

## Introduction

**Fig 1:**
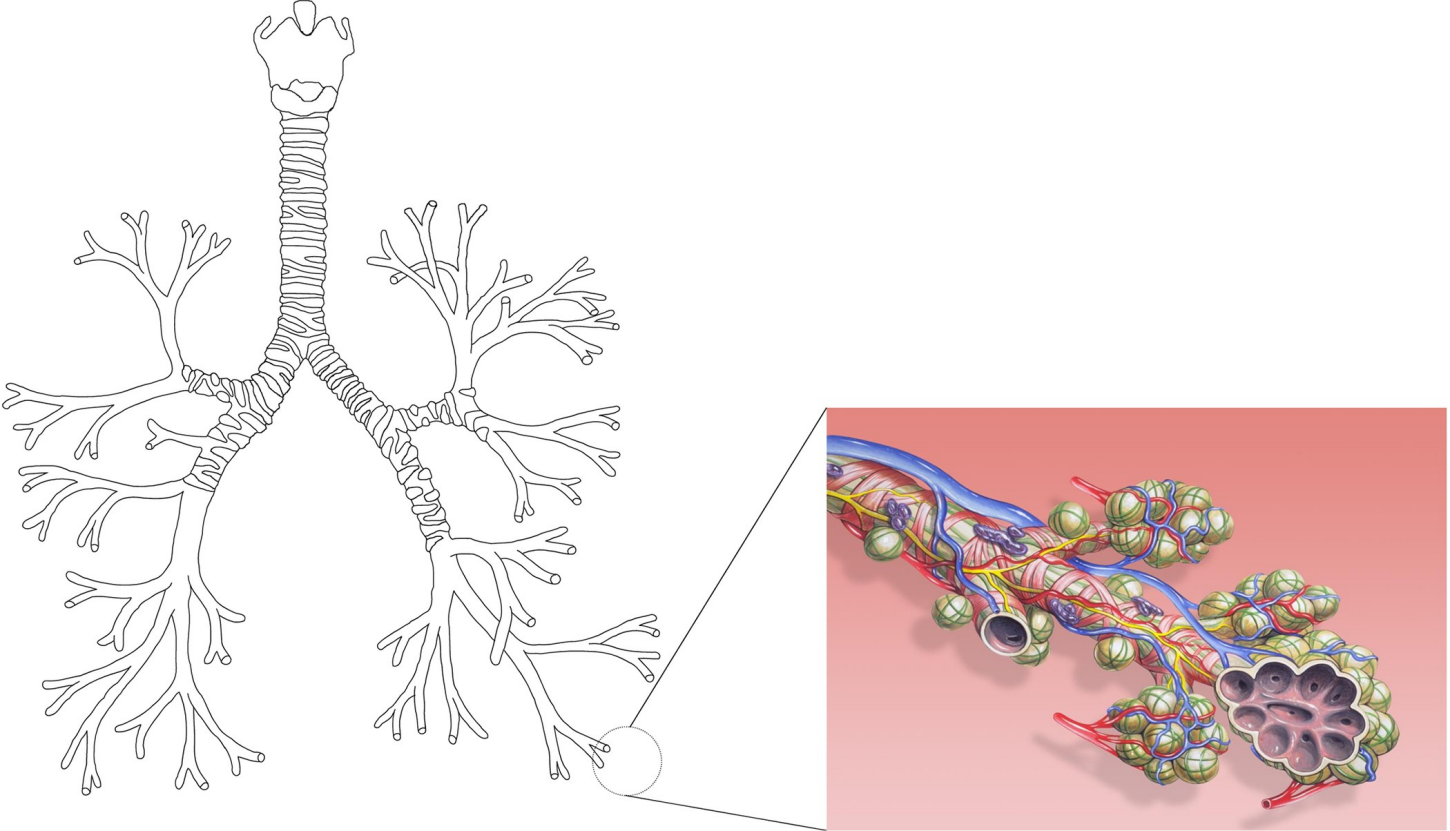
On the left, a schematic representation of the tracheobronchial tree as a dichotomous tubular tree structure (own figure). On the right, a drawing of the alveoli budding from the airway walls of an acinus (Image by Patrick J. Lynch, medical illustrator / Wikimedia Commons. License: Creative Commons Attribution 2.5 License 2006. Bronchial anatomy detail of alveoli and pulmonary circulation).

With over 10,000 liters of air processed a day, the lungs are the body’s major site of exchange with the environment [1]. Their internal structure forms a dichotomous branching tree in which each level of subdivision is called a generation [2] (see Fig 1). The lungs themselves are divided into two regions: the bronchial region, composed of the airways (trachea, bronchi, bronchioles…), and the alveolar region, composed of the acini. A single acinus is usually represented as a seven-generation dichotomous tree structure, in which each generation has an increasing number of alveoli budding from the walls of its airways [2, 3] (see Fig 1). The cells composing the surface of the alveoli form the gas-blood interface, allowing the exchange of dioxygen and carbon dioxide.

The branching structure of the lungs allows the creation of a surface area (between 50 and 100 m^2^) for gas exchange between the body and the environment [3]. To protect the lungs from particles and pathogens, the bronchial epithelium is covered with mucus, produced by goblet cells and other secretory cells in the airway epithelium. Mucus is an aqueous phase that contains several substances, including antibodies, enzymes, and glycoproteins, that help to trap and eliminate inhaled particles and pathogens [4]. Mucociliary clearance is the mechanism by which the bronchial mucus is removed from the lungs. The airway epithelium is lined with specialized cells that have hair-like structures called cilia. The cilia beat in coordinated waves, propelling the bronchial mucus upward toward the pharynx, where it can be swallowed or expectorated [5].

The lungs can be affected by several pathologies, the most common of which is asthma. It is a chronic disease characterized by inflammation and narrowing of the airways, resulting in recurrent episodes of wheezing, breathlessness, chest tightness, and coughing [6]. During an asthma crisis, the airways become inflamed and swollen, leading to increased mucus production. This causes the smooth muscles surrounding the airways to tighten and narrow. This restricts the flow of air in and out of the lungs, causing symptoms such as wheezing and shortness of breath [6]. Inflammation in asthma is typically triggered by exposure to allergens (such as pollen or dust mites), respiratory infections, exercise, or irritants (such as smoke or strong odors) [7], causing the immune system to overreact. Proper management of asthma includes regular medical monitoring and a combination of medications, such as bronchodilators and anti-inflammatory drugs, and lifestyle changes [7]. Another common and severe lung disease is chronic obstructive pulmonary disease (COPD). The Global Initiative for Chronic Obstructive Lung Disease (GOLD) 2024 defines COPD as a heterogeneous lung condition characterized by chronic respiratory symptoms due to abnormalities of the airways (especially narrowing of the small airways less than 2 mm in diameter) and/or the alveoli (emphysema leading to a reduction in the alveolar surface available for gas exchange) [8].

Monitoring lung function in unhealthy patients involves several clinical assessments, including measurement of airway resistance, fraction of exhaled nitric oxide (FeNO), and lung diffusing capacity for nitric oxide (DLNO). Indeed, these parameters provide valuable insight into disease severity. Increased airway resistance is classically associated with bronchoconstriction. FeNO measurement analyzes the concentration of nitric oxide (NO) in a person’s exhaled breath. Elevated FeNO indicates ongoing airway inflammation, which is a hallmark of asthma [9]. DLNO measures the ability of the lungs to transfer NO to the blood, reflecting the functional capacity of the lungs to exchange gases [10]. It helps to assess lung health, particularly in conditions where airway narrowing leads to reduced gas exchange. These measurements are typically made using specialized equipment, such as whole-body plethysmography or impulse oscillometry for airway resistance [11], and FeNO and DLNO analyzers for their respective tests. Regular monitoring of these parameters over time allows clinicians to assess treatment efficacy and adjust therapies as needed, promoting better disease management and quality of life [7, 12].

It should be noted, however, that the results of these tests are not always easy to interpret, as they are the fruit of the interaction of several factors, such as lung geometry, airflow and gas exchange. Medical imaging has emerged as a significant diagnostic tool in assessing lung health/function and can supplement test results [13, 14]. However, the current resolution of these technologies does not allow clear visualization of airways beyond the ninth generation [15, 16]. Nevertheless, mathematical modeling has emerged as a powerful tool for understanding the complex dynamics of the lung function, allowing the exploration of complex phenomena that are difficult to study by traditional experimentation alone. By developing computational frameworks, researchers can simulate (patho)physiological processes, and thus gain a deeper understanding of disease mechanisms. In addition, these models can be used to interpret clinical data, predict treatment outcomes, and guide personalized therapeutic strategies [17].

Most of the models describing airway resistance or the transport and exchange of a gas species such as NO throughout the lungs rely on a homogeneous description of the lung geometry [18–25]. In such a description, all airways belonging to the same generation have the same diameter and length. Such a representation is widely used because of its simplicity, as it allows global consideration of all airways in a single generation. However, this is an idealized representation of the lung geometry, as the bronchial tree has an asymmetric branching pattern: at each bifurcation in the tree, an airway divides into two daughter airways, both of which are smaller than their parent but one of which is larger than the other [26–28]. In addition, in asthma and in other diseases, the lungs may show local inflammation and narrowing of their airways, a feature that obviously cannot be fully accounted for in a model based on a homogeneous description of the lungs.

On the other hand, the number of modeling works considering the whole bronchial tree with the asymmetric nature of its branching pattern is limited [26, 27, 29, 30], and these models are often associated with high computational costs due to the large number of airways considered individually. Furthermore, it is worth noting that several studies have been performed using computational fluid dynamics on airway resistance and gas species exchange in the lungs, considering the asymmetric nature of the bronchial tree. However, these studies often include a subject-specific geometry based on medical imaging and are often limited to a few generations [31–34]. It is also worth mentioning that, to the best of our knowledge, there is no mathematical model that has been developed specifically to simulate a DLNO test.

Based on these elements, the objective of our work is to provide a new computational framework that allows the calculation of airway resistance, FeNO, and DLNO for healthy and unhealthy patients. Our approach is based on a realistic representation of the geometry of healthy lungs as a function of body mass. The original way in which this geometry is created, including an individual definition of the airways in the first seven generations of the lungs, makes it possible to account for the heterogeneous nature of the lungs in terms of perfusion and ventilation. Moreover, the geometry can be easily modified to simulate different abnormalities, local or global (constriction, inflammation, perfusion defect). The computational framework includes the possibility to simulate, on a given (possibly modified) geometry, a test to measure the flow resistance of the lungs (including its component due to the not fully developed flow in the first generations of lungs), a test to measure the concentration of nitric oxide in the exhaled air, and a test to measure the diffusion capacity for nitric oxide. This is implemented in the framework by solving different transport equations (momentum and convection/diffusion) describing these tests.

In this paper, the computational framework is first briefly presented (and fully detailed in the Methods section). It is also provided as supporting information in Python (S1 File) and Wolfram Mathematica (S2 File) files. The computational framework is then used to calculate airway resistance, FeNO, and DLNO under various conditions on healthy and pathological adult lungs. The results are compared with available experimental data to assess the quality of our model and to show that it can provide new insights into lung diseases.

## Summary of the computational framework

The computational framework is presented in detail in the Methods section, with numerous references from the literature to support the modeling choices and the values of the various parameters used. In this section, we give an abridged description of the framework to understand its main elements.

The first function of the computational framework is to create the lung geometry. This geometry is made dependent on the body mass by using allometric laws to define the values of parameters such as the tracheal diameter or the alveolar size [35].

**Fig 2:**
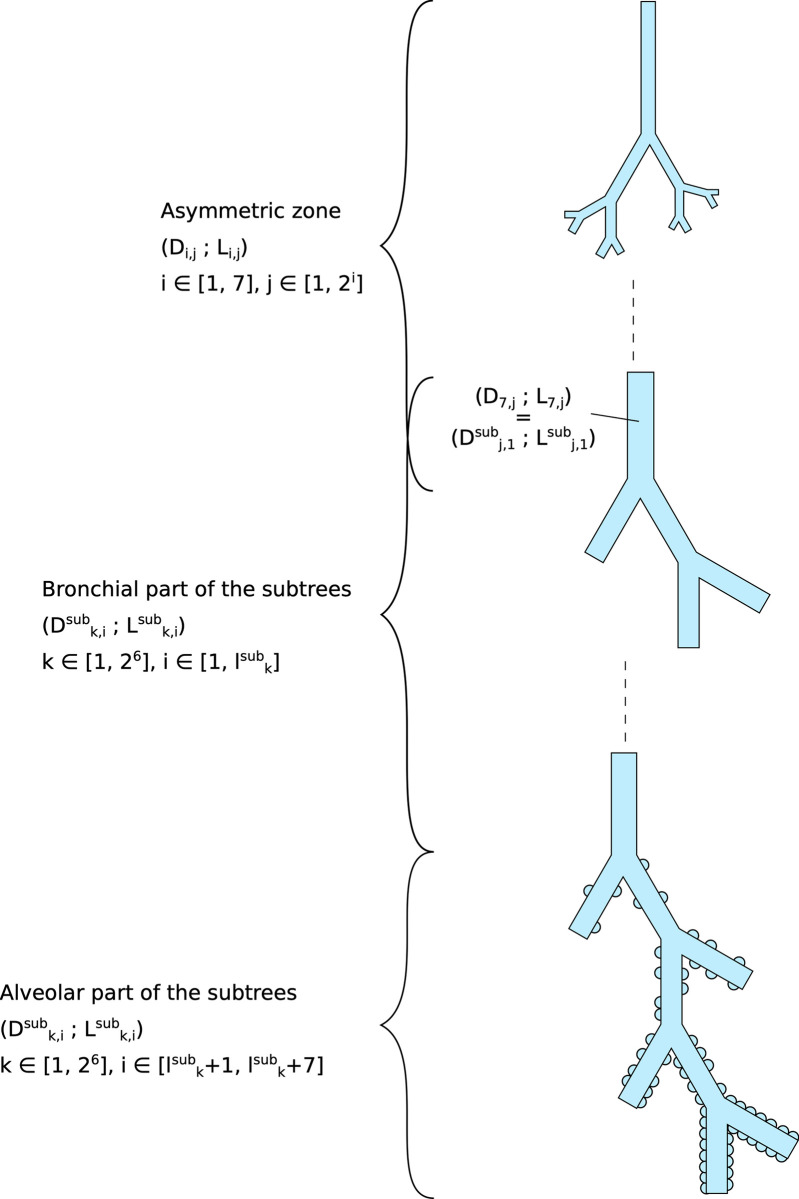
Schematic representation of the geometry of the lungs created by the computational framework.

For a given value of the body mass, the geometry of the lungs is created in two steps (see Fig 2, please note that additional information about the lung geometry and the notations presented in this figure are given in the Methods section).

First, the so-called the “asymmetric zone”, is created. It consists of the first seven generations of the lungs. Within this zone, each airway (i,j) divides into two daughter airways: (i+1,2j-1) and (i+1,2j). Both are smaller than the airway (i,j), but the airway (i+1,2j) is larger than the airway (i+1,2j-1), a feature that is reported in the literature [28]. Since the dimensions of the airways in the first generations of the lungs can be characterized by imaging techniques, data are available in the literature to support the definition of this asymmetric zone [27, 28].

It is important to note that the natural variability of lung geometry from one individual to another is introduced into the computational framework, by defining certain parameters characterizing this asymmetric zone as random variables. So, for a given value of body mass, each time the computational framework is used to create a lung geometry, this geometry is unique and therefore different from geometries created for the same mass.

Then, each of the 64 airways that make up the first seven generations of the lungs, which are all different, is the starting point of what we call a “subtree”. The geometry of these subtrees is more idealized than that of the first seven generations, but it is still based on literature data. Each subtree is a dichotomous homogeneous tree: all the airways are divided into two and all the airways belonging to a same generation of the subtree have the same dimensions. Each subtree consists of two parts: a so-called bronchial part and an alveolar part. Within the bronchial part, the diameter of the successive airways is multiplied by a factor of 0.8 [20]. The bronchial part is stopped if an additional division would have produced airways with a diameter less than twice the alveolar diameter [21]. The alveolar part of a subtree is then composed of 7 additional generations, with airways all having the same diameter and length (common to all subtrees) [2, 18]. Alveoli are budding on the wall of the airways in the alveolar part, in a number defined for each generation based on the morphometric data reported by Weibel [2, 18]. Since the first airways of the different subtrees have different diameters, it follows that the bronchial parts of the subtrees have different numbers of generations. Therefore, the model considers a certain distribution of the number of generations between the trachea and the end of the acini.

Once a lung geometry has been created, it can be modified to simulate diseased lungs. In particular, the diameter of each airway (i,j) in the asymmetric zone can be modified individually, which makes it possible to simulate a constriction. The diameter of all the airways of a given generation of a subtree can also be modified.

A second function of the computational framework is to compute, for a given value of the inspiratory or expiratory flow rate, the flow distribution in a previously defined lung geometry, as well as the corresponding alveolar pressure Δ*P* and total flow resistance. It is important to emphasize that we only evaluate the pressure difference between the trachea and the alveoli due to viscous losses. Nevertheless, we consider the not fully developed flow in the first generations of lungs, due to the high values of Reynolds number, which leads to entrance lengths that are not negligible compared with (or even greater than) the length of the airways. This results in increased velocity gradients at the wall and thus higher viscous losses than in Poiseuille flow [23].

A third function of the computational framework is to calculate the volume fraction (in ppb) of NO in the exhaled air (the so-called FeNO: fraction of exhaled nitric oxide), for a given pulmonary geometry and for a given expiratory flow rate (typically 3 l/min [36]). NO is produced in the bronchial epithelium and can then diffuse into the lumen of the airways. When generating a lung geometry, we assign to each airway a transfer flux of NO, written *J*_NO_, which gives the number of moles of NO transferred from the epithelium of that airway to the lumen, per unit of time and per unit of area of the ASL-lumen interface in the airway. We first give a reference value for this flux, derived from [37] and given in the Methods section, which is the same for all airways. It can then be modified individually for each airway (i,j) of the asymmetric zone or for all the airways of a given generation of a subtree, in order to simulate local or global inflammation in the lungs (increased NO production).

Once a geometry has been created (possibly modified to induce constrictions) and the NO transfer fluxes have been defined, we first calculate the flow distribution within this geometry for the chosen expiratory flow rate. We then calculate the NO concentration field, and thus the FeNO, which is the concentration at the top of the trachea, by solving convection / diffusion transport equations.

Blood has a high affinity for NO. Therefore, NO is largely consumed in the alveoli, where it has a low equilibrium concentration with the blood (about 2 ppb [18]). This results in a diffusive flux of NO toward the end of the acini even during exhalation, which is called back-diffusion [25]. The equations describing the transport of NO in the alveolar part of the subtrees during a FeNO test must therefore include a term that accounts for the consumption of NO by the blood in the alveoli. Since not all the alveoli in the lungs are perfused in the same way, we assign a perfusion coefficient to each subtree, ranging from 0 (no perfusion, and therefore no consumption of NO by the alveoli / no equilibrium concentration) to 1 (maximum perfusion). The values of this coefficient are based on the classical 3-zone model [38]: no perfusion in the upper part of the lungs, maximum perfusion in the lower part, and a transition zone between both. This coefficient can be subsequently modified and set to zero in certain subtrees, for example to simulate emphysema in a patient with COPD.

Finally, the computational framework allows the simulation of a diffusing capacity of the lungs for nitric oxide (DLNO) test, again by solving equations describing the transport of NO in the lungs and its consumption in the alveoli. A DLNO test begins with the rapid inhalation of a given volume of gas (about 4 l), in a short time, typically less than 1 second [39]. The inhaled gas contains NO, at a concentration of about 40 ppm. After inhalation, there is a breath-hold phase of a few seconds, during which the inhaled NO diffuses into the lumen of the lungs and is consumed in the alveoli. Finally, a rapid exhalation is performed and the amount of NO in the exhaled air is measured [40]. The DLNO is then calculated as [41]

$$\mathrm{DLNO}=\frac{V^{\mathrm{insp}}(\mathrm{Log}(C_{\mathrm{in}})-\mathrm{Log}(C_{\mathrm{out}}))}{t^{bh}(P_{\mathrm{atm}}-P_{\mathrm{sat},H_{2}O})}$$ Eq 1

with *V*^insp^ the inhaled volume, *t*^*bh*^ the breath-hold duration, *C*_in_ the inlet NO concentration, *C*_out_ the NO concentration in the exhaled air, *P*_atm_ the atmospheric pressure and $P_{\mathrm{sat},H_{2}O}$ the saturation pressure of water at body temperature.

## Results and discussion

In this section, we demonstrate the use of our computational framework in different situations. Our goal is to show that results from the literature can be retrieved (for healthy or unhealthy lungs), to support the quality of our approach. We also show that, for diseased lungs, our model can shed new light on clinical data from the literature. Regarding body mass, it should be noted that in Belgium the average value for adult females is about 70 kg and the average value for adult males is about 80 kg [42], giving an average body mass of 75 kg for the entire adult population.

### Lung geometry

**Fig 3:**
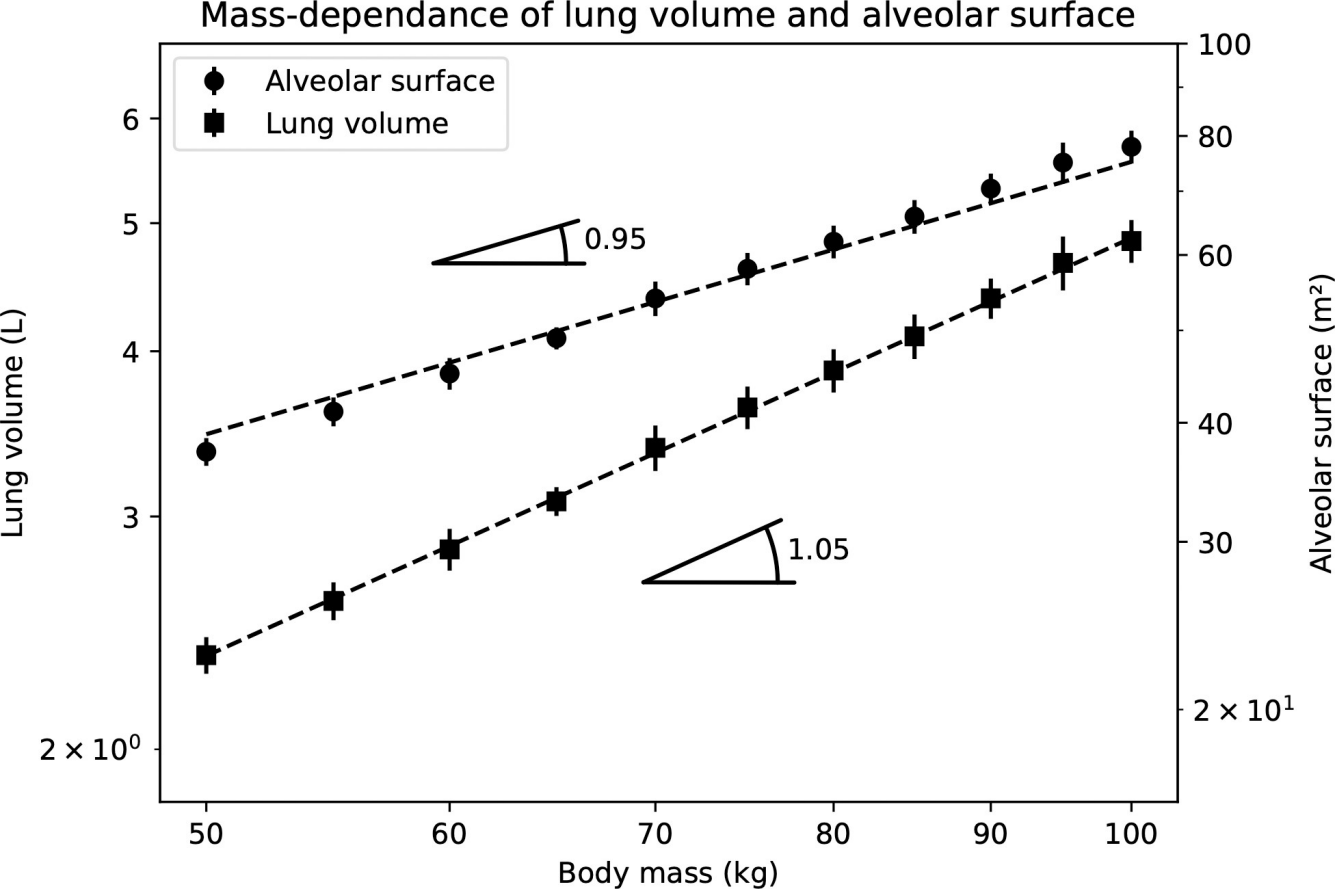
Total lung volume and total alveolar surface area as functions of the body mass, calculated with our model. The dashed lines are the allometric scaling laws reported in [35].

Fig 3 shows the total lung volume (*V*_*t*_), calculated with Eq 6 (see the Methods section), and the total alveolar surface area (*S*_alv_), calculated with Eq 8 (see the Methods section), as functions of the body mass *M*. To generate this figure, 30 lung geometries are created sequentially for each value of *M*, and the means and standard deviations of the resulting lung volume and total alveolar surface area are evaluated. The dashed lines give the allometric laws for *V*_*t*_(∝*M*^1.05^) and for *S*_alv_(∝*M*^0.95^) mentioned by West et al. [35]. The values obtained for *V*_*t*_ and *S*_alv_ are in good agreement with those reported in the literature [2, 18, 43], and we see that our model correctly captures the observed mass dependence of these two global quantities. The calculated values of *S*_alv_ are in the lower range of those reported in the literature. However, it should be noted that this underestimation should be partially compensated, in terms of the NO exchange between the lumen and the blood, by the fact that only part (about 70%) of the surface of an alveolus is covered by capillaries, a feature not considered in our model.

**Fig 4:**
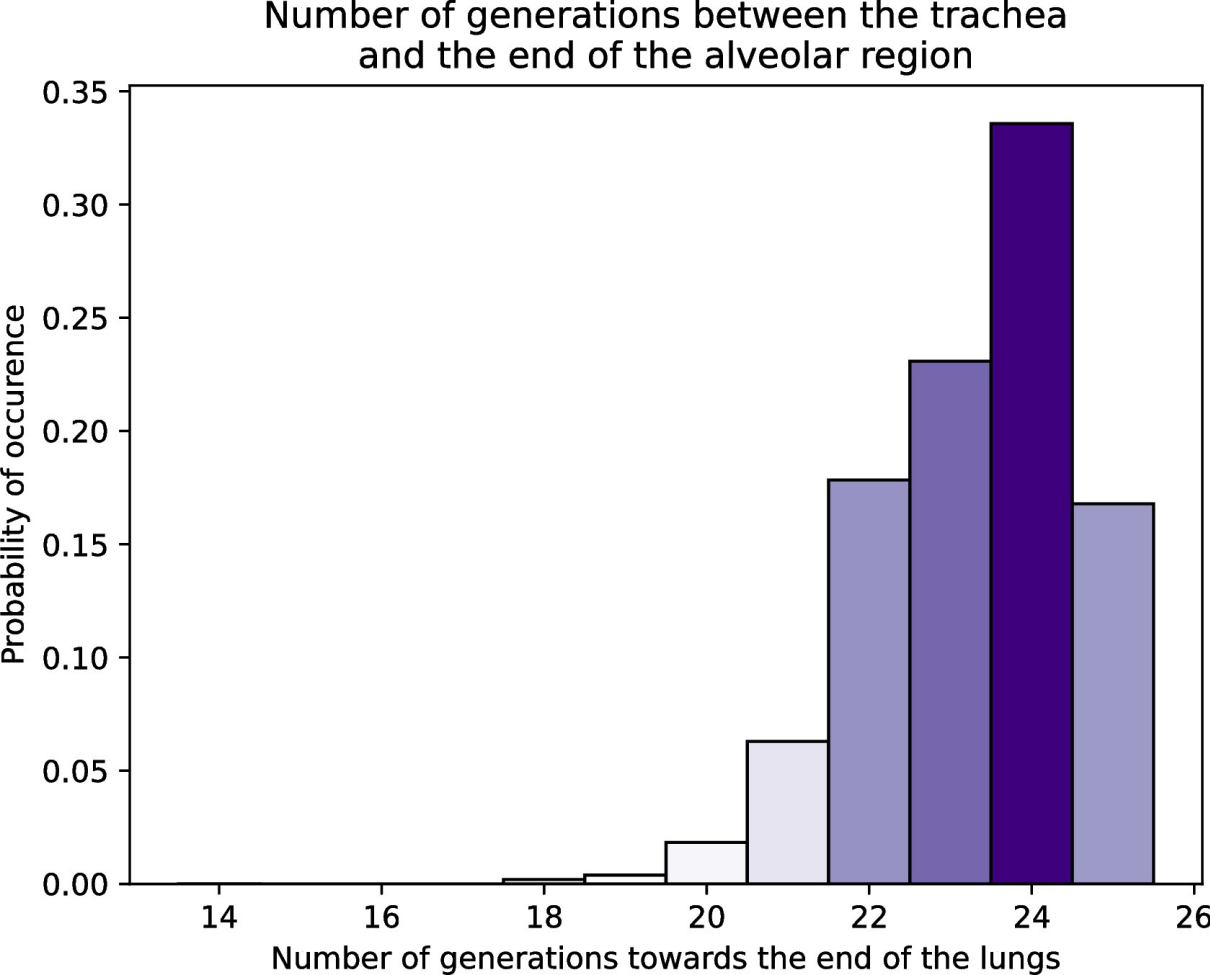
Histogram of the number of generations between the top of the trachea and the end of the alveolar region, averaged on 30 lung geometries created for a body mass of 75 kg.

Fig 4 shows a histogram of the calculated number of generations between the trachea and the end of the alveolar region, averaged over 30 lung geometries created for a body mass of 75 kg. This distribution is also consistent with data reported in the literature [2], with notably a peak at a generation number of 24.

### Resistance to flow

**Fig 5:**
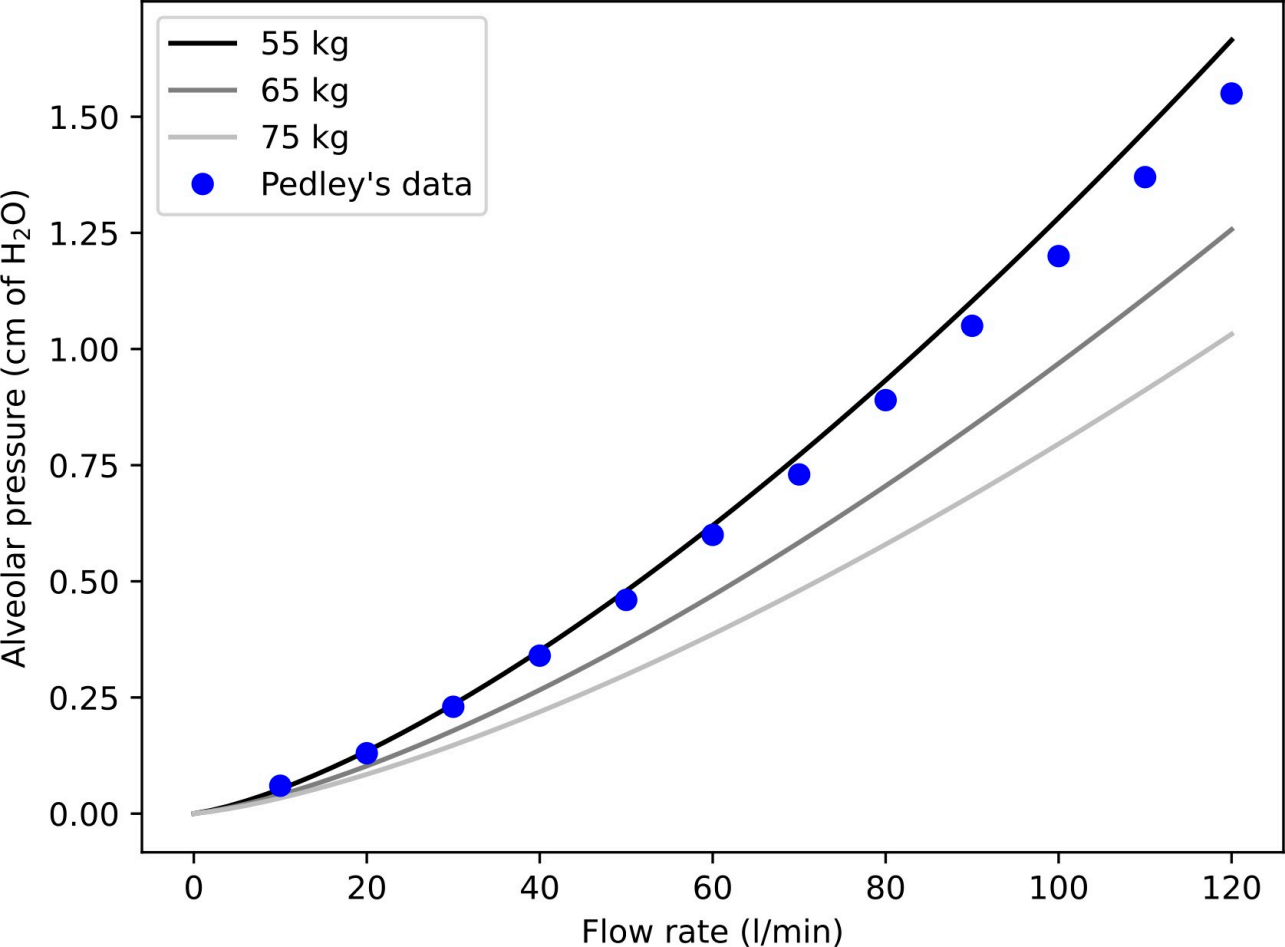
Alveolar pressure Δ*P* (in cm of H_2_O) as a function of the inspiratory flow rate (in l/min), for lung geometries produced with body masses of 55 kg, 65 kg, and 75 kg. The data given by Pedley et al. [23] are also presented in this figure, to show that a similar order of magnitude is recovered.

Fig 5 shows the calculated alveolar pressure Δ*P* (in cm of H_2_O) as a function of the inspiratory flow rate (in l/min), for three lung geometries produced for a body mass of 55 kg (light adult female), 65 kg, and 75 kg (average adult population), respectively. Recall that Δ*P* (given by Eq 14 in the Methods section) is strictly speaking the difference between the pressure at the top of the trachea and the alveolar pressure, due only to viscous losses. The data given by Pedley et al. are also presented in Fig 5 [23]. It should be noted that the three calculated curves are almost insensitive to the natural variation of the geometry (i.e., if they are recalculated based on other lung geometries produced for the same body masses, the new curves do not differ significantly from the ones presented). We also see that these curves are quite sensitive to body mass. The order of magnitude obtained by Pedley et al. is recovered. The non-linear nature of the relationship between alveolar pressure and flow rate clearly shows that the not fully established flow in the first generations has a significant influence on the resistance to flow in the lungs (see Eq 11 and Eq 12 in the Methods section). We can estimate that, at rest, considering this not fully established flow doubles the total resistance to flow compared to a Poiseuille flow and, during exercise, it can even be multiplied by four.

**Fig 6:**
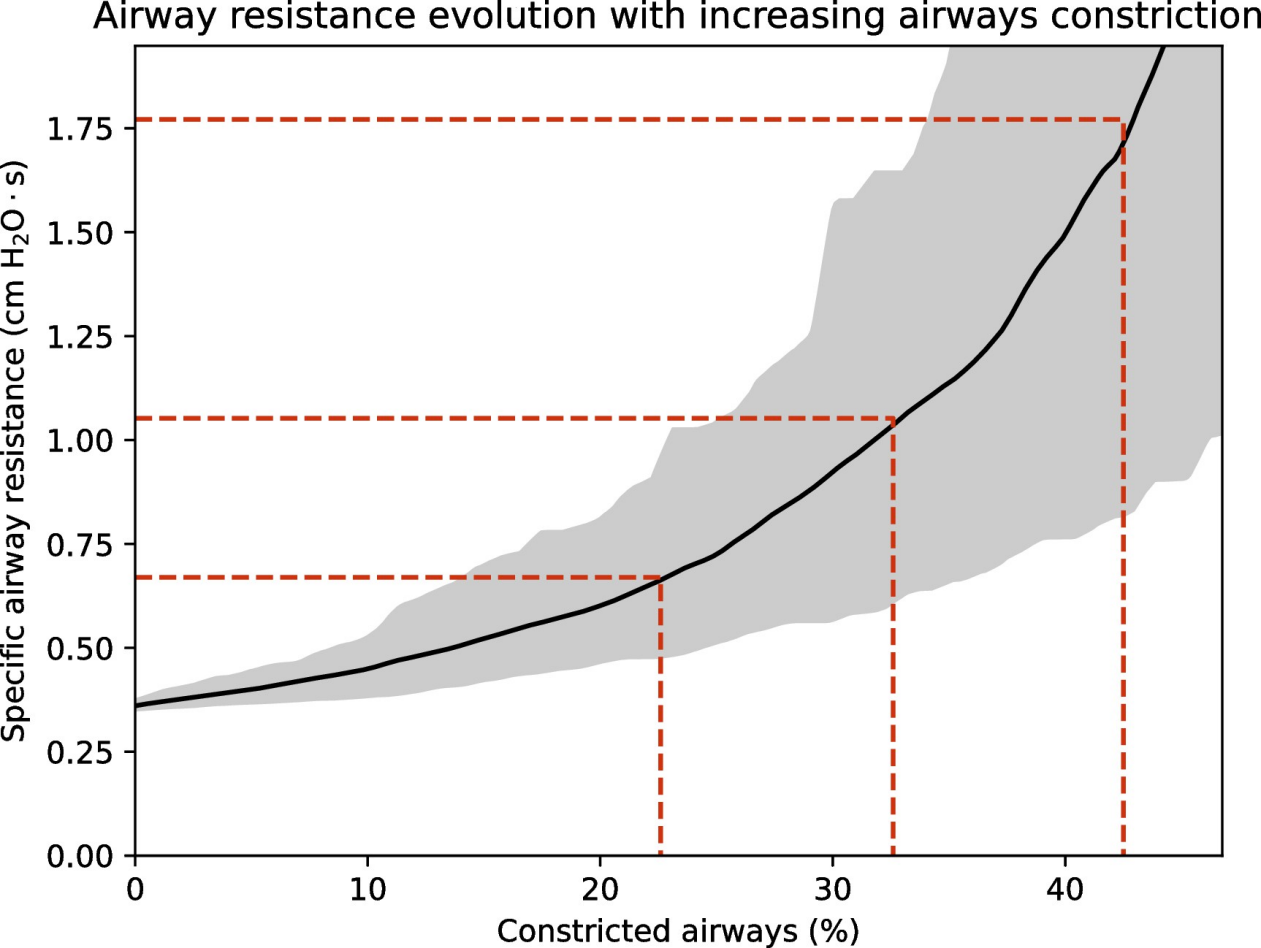
Specific airway resistance, as a function of COPD severity, defined here as the percentage of the small airways (diameter less than 2 mm) affected by constriction, for a body mass of 75 kg. The shaded area gives the 95% confidence interval, reflecting the fact that, for a given body mass, there is a natural variation in lung geometry, and that the same percentage of constriction does not always correspond to the same choice of airways.

Fig 6 shows the calculated evolution of a clinical parameter, the specific airway resistance, as a function of COPD severity, defined here as the percentage of the small airways affected by constriction. Small airways are defined as airways with a diameter of less than 2 mm but excluding the airways in the alveolar parts of the subtrees [44]. This specific airway resistance is

$${sR}_{aw}=\frac{V^{\mathrm{insp}}\Delta P}{Q^{\mathrm{insp}}}$$ Eq 2

with *V*^insp^ and *Q*^insp^ the inhaled volume and inspiratory flow rate during the test, respectively. They are set equal to 1.2 l and 30 l/min, as in [45]. Note that in the usual definition of the specific airway resistance, *ΔP* is the total pressure drop along the respiratory tract. Here, as mentioned above, we take *ΔP*, calculated by Eq 14 in the Methods section, as the difference in pressure between the trachea and the alveolar sacs due to viscous losses only.

To generate the curve in Fig 6, a single body mass of 75 kg is considered. First, a lung geometry is created and then an increasing number of small airways are randomly selected. The diameter of these airways is reduced by a factor of 10, leading to a factor 10,000 increase in their resistance to flow. *sR*_*aw*_ is evaluated for each constriction extent thus created. This procedure is repeated 100 times, and we take the average of the curves *sR*_*aw*_ = *f*(% of small airways constricted) obtained, which differ in terms of the geometries initially created and the airways then randomly selected. Finally, the resulting curve is smoothed by a 10-point rolling average. The shaded area shows the 95% confidence interval.

Fig 6 shows three thresholds for *sR*_*aw*_, each derived from data presented in [46], and corresponding to increasing severity thresholds of COPD (GOLD I, II, and III/IV). The corresponding percentages of constricted small airways are 22.5%, 34.4%, and 43.1%, respectively. These results are consistent with experimental data reported in the literature [44], although they are slightly higher, as these authors report 21% of constriction for GOLD I/II and 35% for GOLD III/IV. This is probably because we only attribute the increase in the pressure difference between the trachea and the alveoli to small airway constrictions, whereas emphysema, which is not modeled here, also contributes to the increase in pressure through the stiffening of the alveolar membrane it causes.

Fig 6 shows that dyspnea in COPD is non-linearly related to the severity of the disease, which may remain hidden for a long time before causing symptoms that rapidly worsen. In healthy lungs, it is essentially the first generations that contribute to overall flow resistance, due to the not fully developed flow that occurs there (high Reynolds numbers). Constriction of the small airways may therefore initially have little effect on this resistance.

### Fraction of exhaled nitric oxide

**Fig 7:**
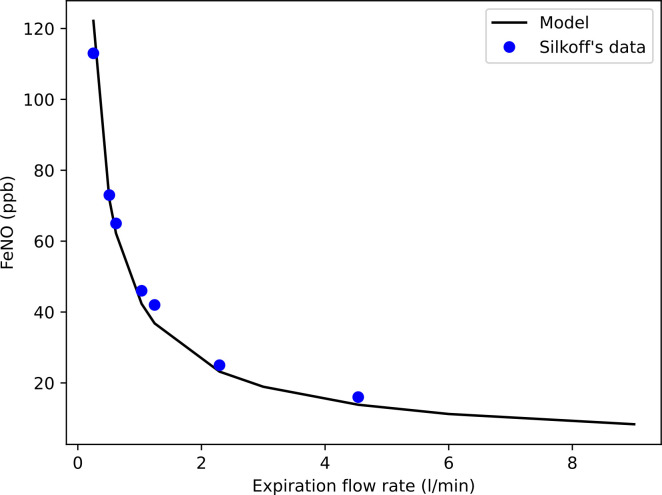
FeNO as a function of the expiratory flow rate, calculated on a single lung geometry obtained for a body mass of 80 kg. The blue dots are the experimental data given by Silkoff et al. [47].

Fig 7 shows the FeNO (in ppb) as a function of the expiratory flow rate, calculated on a single lung geometry obtained for a body mass of 80 kg (around the average value for adult males). The NO transfer flux *J*_*NO*_, which expresses the amount of NO produced in the epithelium of an airway and transferred to its lumen, is taken to be equal to its reference value (given in the Methods section) for all airways. It should be noted that the curve obtained is almost insensitive to natural variations in geometry. The data of Silkoff et al. (average over 10 adult subjects, including 8 males) are also shown in Fig 7 [47].

**Fig 8:**
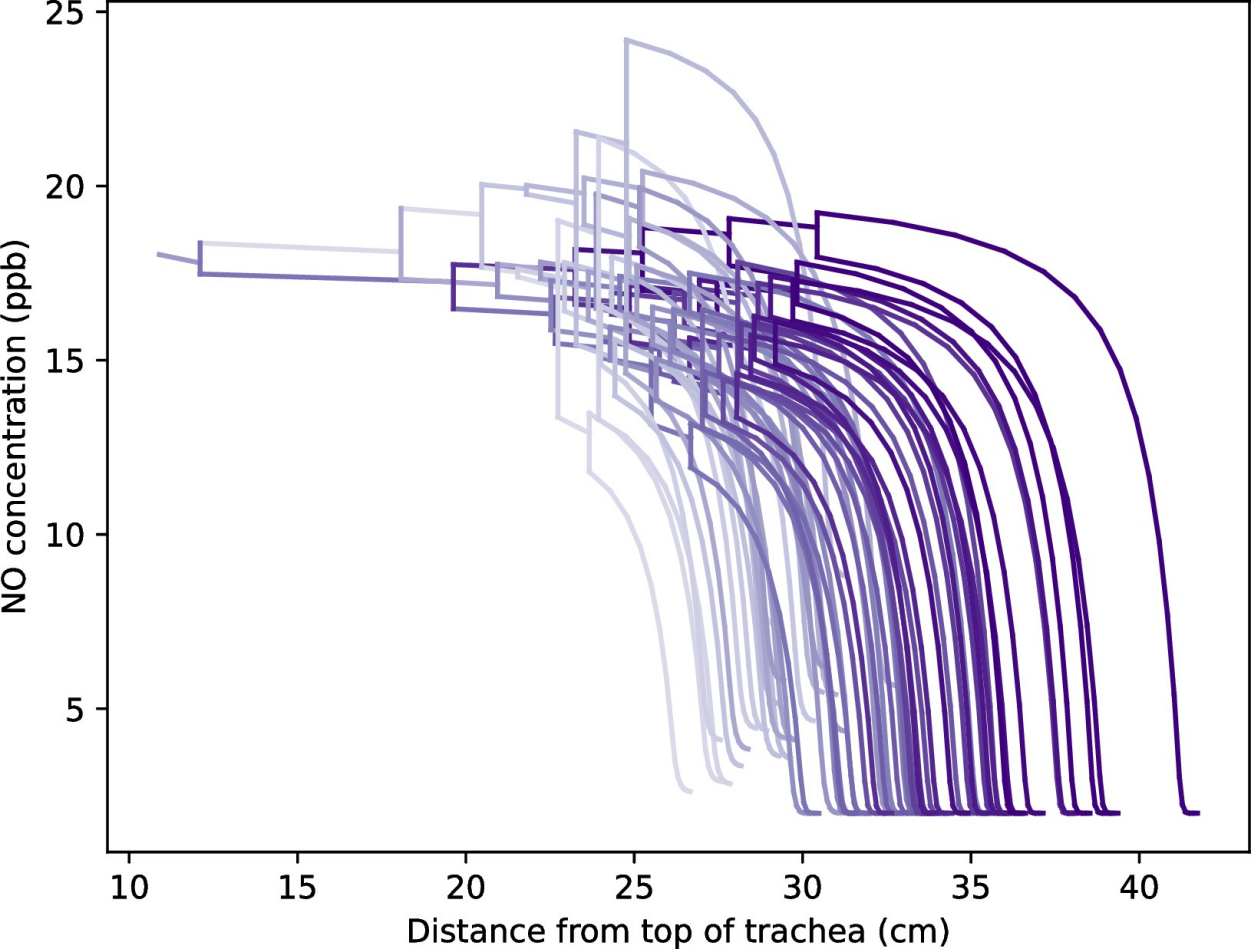
NO concentration profile along the different pathways toward the end of the lungs, at stationary state during a FeNO test with an expiratory flow rate of 3 l/min, on a geometry created for a body mass of 75 kg.

Fig 8 shows the profile of NO concentration along the different pathways to the end of the lungs, at stationary state during a FeNO test with an expiratory flow rate of 3 l/min (a standard value for a FeNO test), on a geometry created for a body mass of 75 kg. The reference value of *J*_*NO*_ is also used here for all airways. In this figure we can clearly see that the NO concentration profile depends on the path followed in the lungs. Within the alveolar part of the subtrees that are perfused, the NO concentration reaches its equilibrium concentration with the blood of 2 ppb. On the other hand, it is logically within the non-perfused subtrees (those closest to the trachea) that we find the highest NO concentrations. The discontinuities observed in the NO concentration profiles occur at the junctions between airways in the asymmetric zone. At this point, two airflows meet during exhalation, and these two flows have a priori different NO concentrations because they have different flow rates and have traveled a different path from the alveolar sacs (i.e., they have received different amounts of NO from the bronchial epithelium). Instantaneous mixing at the junction causes a jump in concentration, which becomes equal to the average of the concentrations in the two flows, weighted by their flow rates.

**Fig 9:**
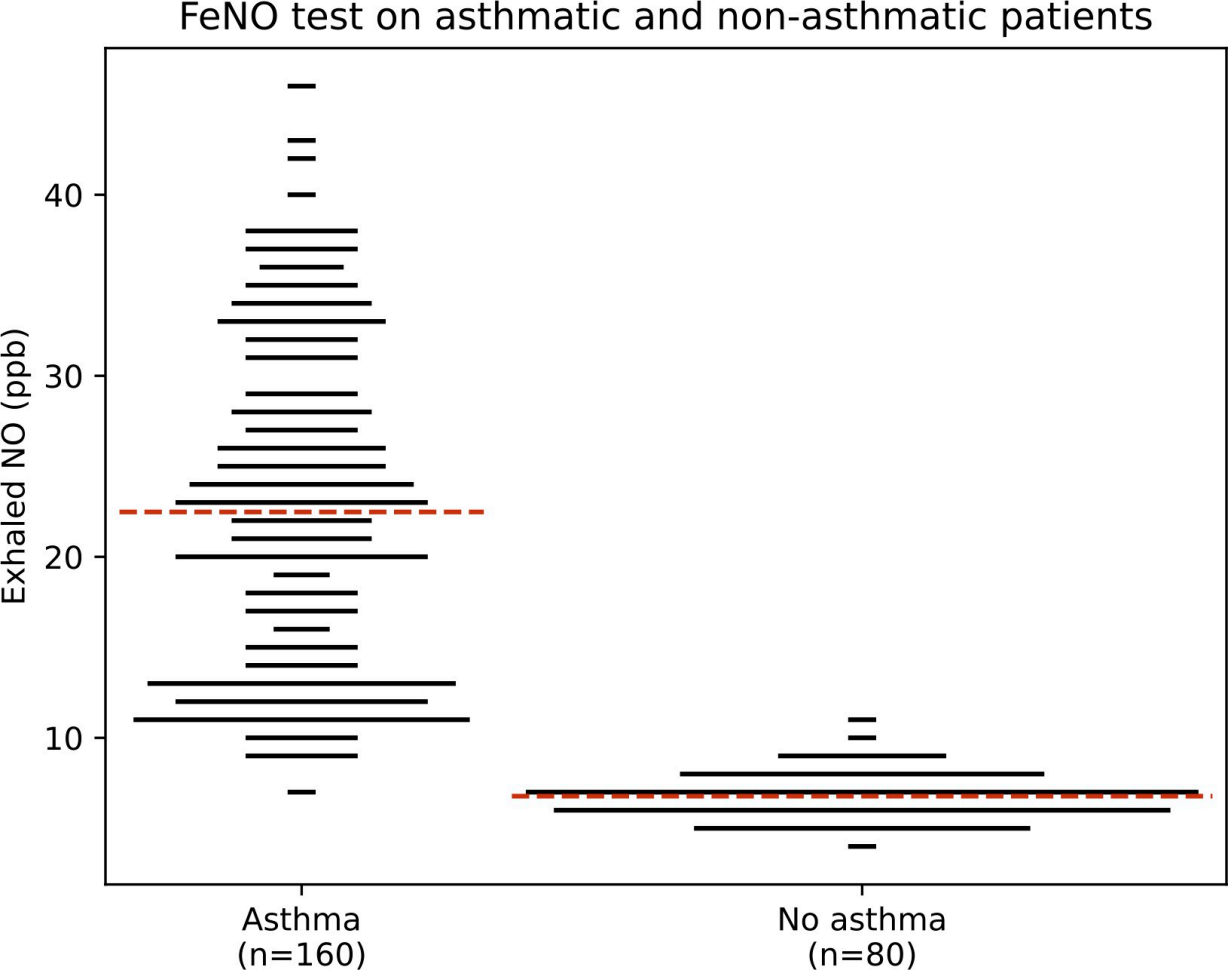
Calculated FeNO values for a population of 80 healthy adults and a population of 160 asthmatics at a fixed expiratory flow rate of 12 l/min. FeNO values are rounded to the nearest ppb and the length of the bars is proportional to the number of people with the corresponding FeNO. The horizontal red dashed lines show the mean values for each population. These results can be compared with those reported by Dupont et al. [48], which provide a similar analysis of the variability of FeNO in healthy and asthmatic adults.

Fig 9 shows the calculated FeNO values for a population of 80 healthy adults and a population of 160 asthmatic adults, at a fixed expiratory flow rate of 12 l/min, such as in [48]. FeNO values are rounded to the ppb and the length of the bars is proportional to the number of people with the corresponding FeNO. The horizontal red dashed lines show the average FeNO value for each population. In each population, we simulate 51% females and 49% males [42]. Before generating its lung geometry, a person’s body mass is randomly selected according to a normal distribution with a mean of 83.3 kg and a standard deviation of 13.9 kg for a man and a mean of 70.4 kg and a standard deviation of 14.3 kg for a woman [42]. In all cases, the NO transfer flux *J*_*NO*_, which expresses the amount of NO produced in the epithelium of an airway and transferred to its lumen, is assumed to be homogeneous in the lungs (i.e., it is the same for all airways). To evaluate the variability of *J*_*NO*_ in healthy subjects, as well as its relative increase in mean value and variability in asthmatic subjects, we use the results presented in [49]. They measured the production of NO by neutrophils, in healthy and asthmatic subjects. They postulated that peripheral blood neutrophils, a type of leukocyte that produces NO in inflammatory reactions [50], being in a primed or activated state in asthma, would reflect the changes that occur in bronchial tree neutrophils. According to their results, *J*_NO_ is assumed to follow a normal distribution independent of mass and sex in healthy subjects, with the reference value of *J*_NO_ as the mean, and a relative standard deviation of 0.16. As for asthmatics, we divide them into three groups: mild asthma, moderate asthma and severe asthma. For each of these groups, and according to the results presented in [49], *J*_NO_ is expressed as follows, independent of mass and sex:

$${\mathrm{Mild}\;\mathrm{asthma}:\;J}_{\mathrm{NO}}=J_{\mathrm{NO},\mathrm{ref}}(1.96+0.3\;X(0,1))$$

$${\mathrm{Moderate}\;\mathrm{asthma}:\;J}_{\mathrm{NO}}=J_{\mathrm{NO},\mathrm{ref}}(4.22+0.375\;X(0,1))$$

$${\mathrm{Severe}\;\mathrm{asthma}:\;J}_{\mathrm{NO}}=J_{\mathrm{NO},\mathrm{ref}}(5.89+0.66\;X(0,1))$$

with *J*_NO,ref_ the reference value of *J*_NO_ (given in the Methods section), and *X*(0,1) the outcome of a normal random variable, centered on 0 and with a standard deviation of 1. Finally, to define the group of asthmatics to which a subject belongs, we consider that, according to [51], 33% of asthmatics suffer from mild asthma, 38% from moderate asthma, and 29% from severe asthma. To simulate the case of an asthmatic patient, we first randomly generate a number x between 0 and 1. If x < 0.33, the patient is considered to have mild asthma, if 0.33 < x < 0.71, the patient is considered to have moderate asthma, and if x > 0.71, the patient is considered to have severe asthma.

The results presented in Fig 9 are very close to the experimental results presented by Dupont et al. [48]; this gives good confidence in the capability of our computational framework. The full data behind the results presented in Fig 9 show that in healthy individuals, the calculated dispersion of FeNO values is similarly due to variability in body mass (independent of gender) and variability in *J*_NO_ value, each contributing to a relative variability of FeNO of about 5%. In the overall asthma population, it is largely the severity of the disease (mild, moderate or severe asthma) that implies differences in FeNO values, making FeNO a good indicator for patient follow-up.

**Fig 10:**
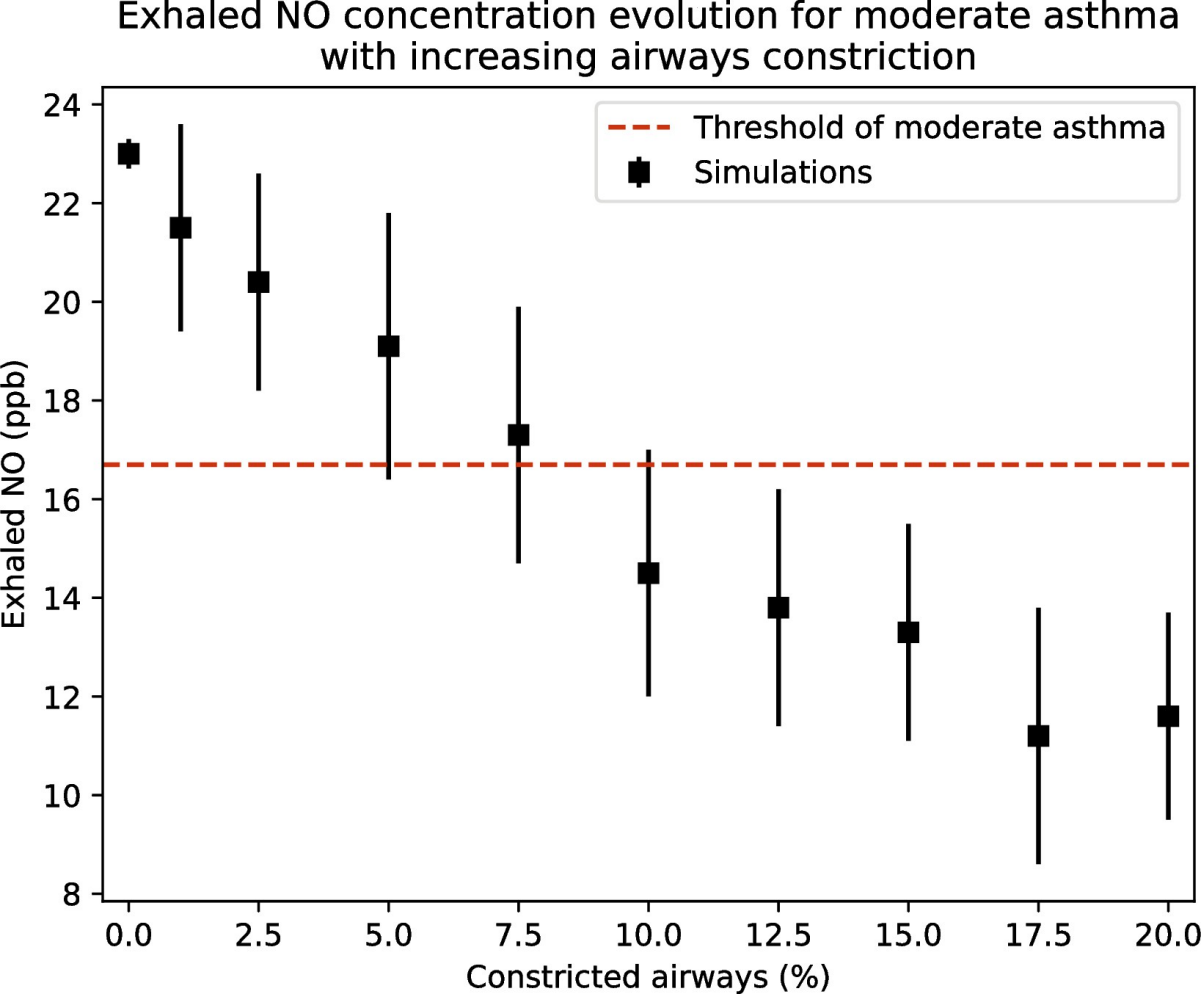
FeNO as a function of the percentage of the bronchial airways affected by constriction. Body mass of 75 kg, expiratory flow rate of 12 l/min. The horizontal dashed line gives the limit between moderate and light asthma.

Finally, Fig 10 shows the effect of increasing numbers of constrictions in the bronchial airways (i.e., all the airways except those in the alveolar parts of the subtrees), which is also a feature of asthma, on the FeNO of mild asthmatics. A constant body mass of 75 kg and an expiratory flow rate of 12 l/min, such as in [48], are considered. The NO transfer flux *J*_NO_ is assumed to be homogeneous in the lungs and equal to 4.22 times its reference value given in the Methods section (average value for moderate asthma). For each value of the percentage of small airways affected by constriction, 30 healthy lung geometries are first created. Then, on each of these geometries, bronchial airways are randomly selected in a number corresponding to the value of the percentage of these airways affected by constriction, and their diameter is reduced by a factor of 10 (resulting in a 10,000-fold increase in their resistance to flow). FeNO is then calculated on the resulting geometry. Finally, the mean and standard deviation of the FeNO values over the 30 geometries are evaluated.

In Fig 10, the dashed horizontal line indicates a limit between mild and moderate asthma. It is evaluated as the average between the third quartile of FeNO values calculated to generate the data in Fig 9 for people with mild asthma and the first quartile of these values for people with moderate asthma. Interestingly, the results presented in Fig 10 show that, when more than about 5% of the bronchial airways are constricted, FeNO is reduced to such an extent that people with moderate asthma could be misclassified as having mild asthma. This is because these constrictions block the transport of NO produced in the airway epithelium to the top of the trachea and promote its back diffusion to the alveoli where it is consumed, resulting in lower values of FeNO. Such an effect of bronchoconstriction on FeNO was highlighted by Michils et al. [36], who reported a decrease in FeNO of 4 ppb for every 10% decrease in forced expiratory volume in one second due to bronchoconstriction.

### Diffusion capacity for nitric oxide

For a male adult with a body mass of 80 kg, DLNO = 215 ml/(min.mmHg) is calculated according to Eq 1 for an inhaled volume of 4 l, an inspiratory time of 0.5 s, and a breath-hold duration of 3 s (average value over 30 lung geometries created for the same body mass). For a breath-hold time of 5 s, DLNO = 129 ml/(min.mmHg) is similarly obtained. This agrees with the results presented in [40] for young male adults.

**Fig 11:**
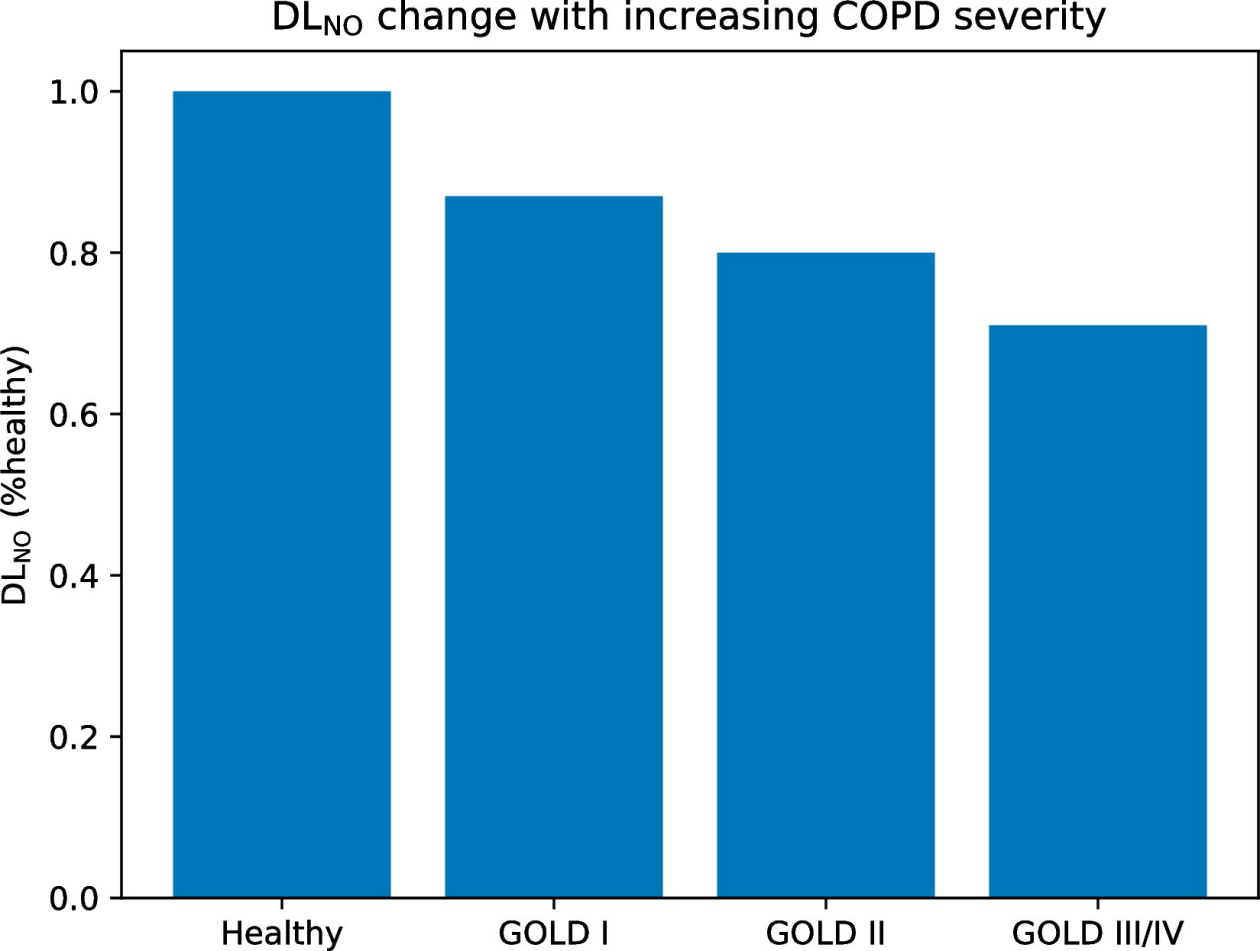
Calculated relative variation of DLNO as a function of the severity of COPD.

In Fig 11, we analyze the effect of COPD severity on the calculated values of DLNO. This figure is obtained at a constant body mass of 75 kg, an inhaled volume of 4 l, an inspiratory time of 0.5 s, and a breath-hold duration of 5 s. Each level of COPD severity (GOLD 1, GOLD 2, and GOLD 3/4) is characterized by a percentage of the small airways (diameter less than 2 mm, excluding the airways in the alveolar part of the subtrees) that are constricted (diameter divided by a factor of 10). These percentages are derived from the results shown in Fig 6: GOLD 1: 22.5% of small airways are constricted, GOLD 2: 34.4%, GOLD 3/4: 43.1%. COPD is also characterized by emphysema, which is simply simulated by setting the perfusion coefficient to zero in some subtrees, thus preventing any NO consumption by blood in these subtrees. According to the data presented in [52], GOLD 1 is characterized by emphysema affecting 3% of the total alveolar surface, GOLD 2 by 12%, and GOLD 3/4 by 22%. To generate Fig 11, a lung geometry is first created. The DLNO value for the healthy situation is calculated on this geometry. GOLD 1 severity is then produced by constricting the appropriate number of small airways (randomly selected) and setting the perfusion coefficient to zero in several randomly selected subtrees, which together represent an alveolar surface area of approximately 3% of the total alveolar surface area. Note that if a subtree whose perfusion coefficient is already zero (because it is in the “no perfusion” zone of the lungs) is randomly selected, its perfusion coefficient of course remains zero, but it is counted in the evaluation of the alveolar area affected by emphysema. After applying these modifications to the healthy lungs, the DLNO corresponding to the GOLD 1 situation is evaluated. Then, GOLD 2 and GOLD 3/4 severities are successively produced by increasing the number of constricted airways and subtrees affected by emphysema, and the corresponding DLNO are evaluated. This procedure is repeated 30 times (thus starting with 30 different geometries corresponding to the same body mass), and the average DLNO values corresponding to the healthy state, GOLD 1, GOLD 2, and GOLD 3/4 are evaluated.

The results in Fig 11 show a qualitative comparison with the data presented in [46]. Indeed, these authors report an average value for DLNO at the GOLD 1 stage equal to 78% of the healthy value (versus 87% from the simulations), while it decreases to 65% at the GOLD 2 stage (versus 80% from the simulations), and 57% at the GOLD 3/4 stage (versus 71% from the simulations).

The results also indicate that the decrease in DLNO with increasing severity is mainly due to emphysema, which seems logical. In addition, we can see that the decrease in DLNO appears to be progressive with disease severity. It is therefore a more sensitive marker than flow resistance in the early stages of the disease.

## Conclusion

In this article, we propose a new computational framework to simulate different transport processes in the lungs. It allows to generate a realistic geometry for the lungs, depending on the body mass, and to modify it to consider different abnormalities (airway obstruction, inflammation, perfusion defect). It allows the simulation of different clinical tests (airway resistance, FeNO, DLNO) classically used in the monitoring of patients suffering from different lung pathologies, thus providing additional information that may be difficult to obtain, for example, by imaging. This framework is described in detail in this article, and its relevance is demonstrated through several simulation cases, yielding results that compare very well with data from the literature, both for healthy and diseased lungs. Python (S1 File) and Wolfram Mathematica (S2 File) files describing the framework are provided as supporting information. They allow anyone who wishes to use / modify / reproduce it in another programming language.

Currently, the user of the computational framework only needs to specify the subject’s mass, and the geometry of the lungs is automatically created by the code. Thus, a "standard" geometry is created, which does not allow for the consideration of a patient that is an outlier in term of lung morphology. However, a simple extension of the code could be to allow the user to modify the parameters that define the geometry of the first 7 generations (asymmetric zone), to simulate the different tests for a specific patient. Of course, this requires detailed information on the geometry of the first generations of the patient’s lungs, but this is entirely possible with current imaging techniques [28, 34, 53].

Still about imaging, we know that conventional ventilation tests can give contradictory results, and that dynamic/functional imaging seems to be a more accurate alternative method for characterizing regional ventilation and gas concentration in the lungs [13, 14]. At this stage, the framework is not intended to offer a comparison with the results of such a technique, although this may be an interesting prospect for further development.

## Methods

### Data

The values of the parameters used in the model are presented in Tables 1–3. They were obtained or derived from several references in the literature. As shown in the Results section, the values of the parameters related to the geometry allow to create a realistic representation of the lungs.

**Table 1:** Values of several parameters used in the model.

Parameter	Notation	Value	References
Alveolar diameter, for a reference body mass of 70 kg	*d*_alv,ref_	200 10^−6^ m	[2, 18]
Density of the air	*ρ*	1.14 kg/m^3^	[54]
Diameter of the trachea, for a reference body mass of 70 kg	*D*_1,ref_	1.7 10^−2^ m	[55]
Diameter of the airways in the acini	*D*_*a*_	400 10^−6^ m	[2, 18]
Diffusion coefficient of NO in air (atmospheric pressure of 1 atm)	*D*_NO_	2.17 10^−5^ m^2^/s	[18]
Dynamic viscosity of the air	*μ*	1.9 10^−5^ kg/(m.s)	[54]
Equilibrium NO concentration in the alveoli (atmospheric pressure of 1 atm)	*C*_eq_	2 ppb	[18, 25]
Length to diameter ratio of the airways in the bronchial part of the subtrees, for a reference body mass of 70 kg	*β*_ref_	3.5	[2, 18]
Length of the airways in the acini	*L*_*a*_	900 10^−6^ m	[2, 18]
Mass transfer coefficient of the NO through the alveolar epithelium	*k*_NO_	3 10^−3^ m/s	[18]
NO transfer flux from the bronchial epithelium to the lumen (reference value for a healthy adult, at an atmospheric pressure of 1 atm)	*J*_NO,ref_	2 10^−3^ (ppb.m^3^/(m^2^.s))	[37]
Ratio of the diameters of two successive airways in the bronchial part of the subtrees	*h*	0.8	[20]
Reference body mass	*M*_ref_	70 kg	

**Table 2:** Parameters defining the asymmetric zone of the lungs.

Generation index in the asymmetric zone *i*	Ratio of the diameter of airway (i+1,2j-1) to the diameter of airway (i,j), $h_{i,0}^{\min}$ [27]	Ratio of the diameter of airway (i+1,2j) to the diameter of airway (i,j), $h_{i,0}^{\max}$ [27]	Length to diameter ratio of the airways, for a reference body mass of 70 kg, *α*_*i*,ref_ [2, 18]
1	0.69	0.87	7
2	0.67	0.80	5
3	0.67	0.83	3
4	0.74	0.86	2
5	0.67	0.87	3
6	0.67	0.87	3.5
7	0.8	0.8	3.5

**Table 3:** Number of alveoli in each generation of an acinus. These values were derived from [2, 18].

Generation index *s* in an acinus	Number of alveoli flourishing on a single duct, *n*_alv,*s*_
1	16
2	32
3	42
4	70
5	61
6	75
7	75

### Geometry of healthy lungs

In the computational framework, the airways of the lungs are right circular cylinders and, as mentioned in the introduction, the lungs are represented as a dichotomous branching tree (i.e., each airway splits into two daughters) until the end of the alveolar region. The data defining the lungs geometry are given in Tables 1–3.

As described below and sketched in Fig 2, the geometry of the healthy lungs is created, for a given value of body mass *M*, in two steps. Note that, strictly speaking, when referring to the diameter of an airway, we should rather refer to the diameter of the lumen of that airway. In the following, we will avoid repeating the term “lumen” each time we refer to the dimensions of an airway.

#### Asymmetric zone

The first step is to create an “asymmetric zone”, starting with the trachea. It is composed of seven generations of airways. The goal is to mimic a realistic geometry in terms of airway diameters and lengths, according to data available. Airway (1,1) is the trachea and each airway divides into two daughter airways when progressing into the bronchial tree; the division of (i,j) gives the airways (i+1,2j-1) and (i+1,2j). *D*_*i*,*j*_ is the diameter of the airway (i,j) and *L*_*i*,*j*_ is its length.

The diameter of the trachea depends on the body mass *M* as [35]

$$D_{1,1}=D_{1,\mathrm{ref}}{\left(\frac{M}{M_{\mathrm{ref}}}\right)}^{\frac{3}{8}}$$

- with *D*_1,ref_ the value of the diameter of the trachea at the reference body mass *M*_ref_ = 70 kg, given in Table 1.

Each airway in the first 6 generations of the asymmetric zone thus divides into two daughter airways. The literature shows that both these daughter airways are smaller than their parent but one of them, called the minor airway, is smaller than the other, called the major airway. We define airway (i+1,2j-1) as the minor daughter of airway (i,j) and airway (i+1,2j) as its major daughter. The ratio of the diameter of airway (i+1,2j-1) to the diameter of airway (i,j) is written $h_{i,j}^{\min}$, while the ratio of airway (i+1,2j) to the diameter of airway (i,j) is written $h_{i,j}^{\max}$. So, we have

$$\forall i=1,\ldots ,6\;and\;\forall j=1,\ldots ,2^{i-1}:\;\left\{ \begin{array}{c} D_{i+1,2j-1}=h_{i,j}^{\min}D_{i,j}\\ D_{i+1,2j}=h_{i,j}^{\max}D_{i,j} \end{array}\right.$$

Regarding the inherent local anatomical variability, Florens et al. have shown that an anticorrelation between the volumes of the two daughters of a parent airway can at best reproduce the morphometric data reported in the literature [27]. Therefore, for each generation i, we define mean values for the parameters $h_{i,j}^{\min}$ and $h_{i,j}^{\max}$ (independent of body mass), written $h_{i,0}^{\min}$ and $h_{i,0}^{\max}$ respectively, and, while defining the daughters of airway (i,j) when generating a lung geometry, we calculate $h_{i,j}^{\min}$ and $h_{i,j}^{\max}$ as follows:

$$h_{i,j}^{\max}=h_{i,0}^{\max}+\sigma X(0,1)$$

$${\left(h_{i,j}^{\min}\right)}^{3}+{\left(h_{i,j}^{\max}\right)}^{3}={\left(h_{i,0}^{\min}\right)}^{3}+{\left(h_{i,0}^{\max}\right)}^{3}$$

where *σ* is the physiological variability of $h_{i,j}^{\max}$, taken to 0.05 to generate all the results presented in this paper [26, 29], and *X*(0,1) is the outcome of a normal random variable, centered on 0 and with a standard deviation of 1.

The values of $h_{i,0}^{\min}$ and $h_{i,0}^{\max}$ are given in Table 2. Their dependence on i allows creating a realistic morphology of the first generations of the lungs [27, 28].

All the airways in generation i of the asymmetric zone have the same length to diameter ratio. It is written *α*_*i*_ and depends on the body mass *M* as [35]

$$\alpha_{i}=\alpha_{i,\mathrm{ref}}{\left(\frac{M}{M_{\mathrm{ref}}}\right)}^{-\frac{1}{8}}$$

with *α*_*i*,ref_ the values of *α*_*i*_ at the reference mass *M*_ref_ = 70 kg, given in Table 2. *α*_*i*,ref_ depends on i to correctly reproduce the morphology of the first generations of the lungs, with notably the small length to diameter ratio of the airways in the fourth generation [18, 28]. We thus have

$$\forall i=1,\ldots ,7\;and\;\forall j=1,\ldots ,2^{i-1}:\;L_{i,j}=\alpha_{i}D_{i,j}$$

#### Subtrees

In a second step, we create what we call “subtrees”. Each airway in generation 7 of the asymmetric zone is the first airway of a subtree. So we have 2^6^ = 64 subtrees. The airway (7,k) in the asymmetric zone is the first airway of the subtree k. The geometry of these subtrees is more idealized than that of the first generations, but it is nevertheless supported by numerous references in the literature [2, 18, 20–22, 24].

Each subtree is a dichotomous homogeneous tree: all the airways split into two and all the airways belonging to a same generation of the subtree have the same dimensions. The labeling of the generations of a subtree starts again from 1. The diameter and the length of the airways in generation i (≥1) of the subtree k are written $D_{k,i}^{\mathrm{sub}}$ and $L_{k,i}^{\mathrm{sub}}$, respectively.

Since the airway (7,k) in the asymmetric zone is the first airway of the subtree k (see Fig 2), we have $D_{k,1}^{\mathrm{sub}}=D_{7,k},\forall k=1,\ldots ,2^{6}$.

Each of these subtrees consists of two parts: a bronchial part without alveoli and an alveolar part with alveoli (see Fig 2).

The number of generations in the bronchial part of the subtree k is written $l_{k}^{\mathrm{sub}}$. The airways in generations 1 to $l_{k}^{\mathrm{sub}}-1$ of this bronchial part divide into two identical daughters, with a reduction of the diameter by a factor *h* = 0.8, slightly above the theoretical value of 2^−1/3^ given by the Hess-Murray law [20]. Thus we have

$$D_{k,i}^{\mathrm{sub}}={h^{i-1}D}_{k,1}^{\mathrm{sub}},\forall i=2,\ldots ,l_{k}^{\mathrm{sub}}$$

All the airways in the bronchial part of all the subtrees have the same length to diameter ratio. It is written *β* and depends on the body mass *M* as [35]

$$\beta =\beta_{\mathrm{ref}}{\left(\frac{M}{M_{\mathrm{ref}}}\right)}^{-\frac{1}{8}}$$

with *β*_ref_ the value of *β* at the reference mass *M*_ref_ = 70 kg, given in Table 1.

Thus we have

$$L_{k,i}^{\mathrm{sub}}={\beta D}_{k,i}^{\mathrm{sub}},\forall i=1,\ldots ,l_{k}^{\mathrm{sub}}$$

The number of generations in the bronchial part of the subtree k, $l_{k}^{\mathrm{sub}}$, is determined by assuming that the bronchial part stops when an additional division would have produced an airway with a diameter less than twice the inner diameter of the alveoli (written *d*_alv_) [2, 18, 20, 35]. In other words, $D_{k,l_{k}^{\mathrm{sub}}}^{\mathrm{sub}}={h^{l_{k}^{\mathrm{sub}}-1}D}_{k,1}^{\mathrm{sub}}\geq {2d}_{\mathrm{alv}}$ and $h^{l_{k}^{\mathrm{sub}}}D_{k,1}^{\mathrm{sub}}<{2d}_{\mathrm{alv}}$. Therefore:

$$l_{k}^{\mathrm{sub}}=\mathrm{Floor}\left[1+\mathrm{Log}(\frac{2d_{\mathrm{alv}}}{D_{k,1}^{\mathrm{sub}}})\frac{1}{\mathrm{Log}(h)}\right]$$

with Floor[x] the greatest integer less than or equal to x. According to the way the asymmetric zone is created, the diameters of the first airways of the subtrees are not equal. Consequently, $l_{k}^{\mathrm{sub}}$ depends on k. For example, for *M* = 70 kg and if the physiological variability on $h_{i,j}^{\max}$ is set to zero, $l_{k}^{\mathrm{sub}}$ ranges from 7 (k = 1) to 13 (k = 2^6^).

The diameter of the alveoli *d*_alv_ depends on the body mass M as [35]

$$d_{\mathrm{alv}}=d_{\mathrm{alv},\mathrm{ref}}{\left(\frac{M}{M_{\mathrm{ref}}}\right)}^{\frac{1}{12}}$$

with *d*_alv,ref_ the value of *d*_alv_ at the reference body mass *M*_ref_ = 70 kg, given in Table 1.

The alveolar part of a subtree is composed of 7 additional generations, with airways having all the same diameter *D*_*a*_ and length *L*_*a*_ (common to all the subtrees):

$$D_{k,i}^{\mathrm{sub}}=D_{a},\forall i=l_{k}^{\mathrm{sub}}+1,\ldots ,l_{k}^{\mathrm{sub}}+7$$

$$L_{k,i}^{\mathrm{sub}}=L_{a},\forall i=l_{k}^{\mathrm{sub}}+1,\ldots ,l_{k}^{\mathrm{sub}}+7$$

In these seven last generations of the subtrees, alveoli are budding on the wall of the airways. They are represented as hemispheres with diameter *d*_alv_. The number of alveoli budding on the wall of a single airway in generation $l_{k}^{\mathrm{sub}}+s$ of a subtree is written *n*_alv,*s*_. The values of *n*_alv,*s*_ are given in Table 3. They are directly derived from the measurements of the alveolar volume in each generation of an acinus realized by Weibel and reported in [18], considering the alveoli as hemispheres with a diameter of 200 microns.

Like Weibel [18], we can define a “total volume” diameter of the airways in generation i of the subtree k, written ${\bar{D}}_{k,i}^{\mathrm{sub}}$. This “total volume” diameter is such that $L_{k,i}^{\mathrm{sub}}{\pi ({\bar{D}}_{k,i}^{\mathrm{sub}})}^{2}/4$ is the total volume of air in an airway in generation i of the subtree k (i.e., including the volume of air in the alveoli budding on the airway wall).

As there are no alveoli if $i\leq l_{k}^{\mathrm{sub}}$, we have

$${\bar{D}}_{k,i}^{\mathrm{sub}}=D_{k,i}^{\mathrm{sub}},\forall i=1,\ldots ,l_{k}^{\mathrm{sub}}$$

For $i=l_{k}^{\mathrm{sub}}+s(\forall s=1,\ldots ,7)$, we can write that

$$L_{k,i}^{\mathrm{sub}}\pi \frac{{\left({\bar{D}}_{k,i}^{\mathrm{sub}}\right)}^{2}}{4}=\frac{\pi {\left(D_{k,i}^{\mathrm{sub}}\right)}^{2}}{4}L_{k,i}^{\mathrm{sub}}+n_{\mathrm{alv},s}\left(\frac{1}{2}\frac{\pi d_{\mathrm{alv}}^{3}}{6}\right)$$

or

$${\bar{D}}_{k,i}^{\mathrm{sub}}=D_{k,i}^{\mathrm{sub}}\sqrt{1+n_{\mathrm{alv},s}\frac{d_{\mathrm{alv}}^{3}}{3L_{k,i}^{\mathrm{sub}}{\left(D_{k,i}^{\mathrm{sub}}\right)}^{2}}}$$ Eq 3

#### Characteristics of the geometry

Once the geometry of healthy lungs has been created, we can calculate several of its characteristics. The volume of the first 6 generations of the asymmetric zone is

$$V_{1}=\sum_{i=1}^{6}\sum_{j=1}^{2^{i-1}}\frac{\pi }{4}D_{i,j}^{2}L_{i,j}$$ Eq 4

The volume of the subtree k is

$$V_{k}^{\mathrm{sub}}=\sum_{i=1}^{l_{k}^{\mathrm{sub}}+7}2^{i-1}\frac{\pi }{4}{\left({\bar{D}}_{k,i}^{\mathrm{sub}}\right)}^{2}L_{k,i}^{\mathrm{sub}}$$ Eq 5

Therefore, the total volume of the lungs, which is the so-called functional residual capacity (FRC), is

$$V_{t}=V_{1}+\sum_{k=1}^{2^{6}}V_{k}^{\mathrm{sub}}$$ Eq 6

We define the “terminal” airways of the subtree k as the airways in generation $l_{k}^{\mathrm{sub}}$ of that subtree (i.e., these are the airways connected to the alveolar region of the subtree). Since $l_{k}^{\mathrm{sub}}$ is the number of generations in the bronchial part of the subtree k, the number of terminal airways in this subtree is $2^{l_{k}^{\mathrm{sub}}-1}$, and the total number of terminal airways in the lungs is

$$n^{\mathrm{ter}}=\sum_{k=1}^{2^{6}}2^{l_{k}^{\mathrm{sub}}-1}$$ Eq 7

As mentioned above, the alveoli are represented as hemispheres with a diameter written *d*_alv_, and the number of alveoli budding on the wall of a single airway in the generation $l_{k}^{\mathrm{sub}}+s$ of a subtree is written *n*_alv,*s*_. Therefore, the total surface area of the alveoli involved in gas exchange is

$$S_{\mathrm{alv}}=\sum_{k=1}^{2^{6}}\left(2^{l_{k}^{\mathrm{sub}}}\sum_{s=1}^{7}2^{s-1}n_{\mathrm{alv},s}\frac{\pi d_{\mathrm{alv}}^{2}}{2}\right)$$ Eq 8

or

$$S_{\mathrm{alv}}=\frac{\pi d_{\mathrm{alv}}^{2}}{2}\left(\sum_{k=1}^{2^{6}}2^{l_{k}^{\mathrm{sub}}}\right)\left(\sum_{s=1}^{7}2^{s-1}n_{\mathrm{alv},s}\right)$$

Starting from the trachea, the number of generations before reaching the end of the alveolar part of the subtree k is $6+l_{k}^{\mathrm{sub}}+7$, and there are $2^{l_{k}^{\mathrm{sub}}+7-1}$ final airways in the alveolar part of that subtree. This allows plotting a histogram of the number of generations between the trachea and the end of the alveolar region.

The “geom” function in the Wolfram Mathematica file and the “geom” object in the Python file create the geometry of healthy lungs for a given value of the body mass *M* in kg. It also calculates the total volume of the lungs (FRC), the total number of terminal airways *n*^ter^, the average number of airways on the path to the alveolar region (average of $6+l_{k}^{\mathrm{sub}}$), the total surface area of the alveoli, and the histogram of the number of generations between the trachea and the end of the alveolar region.

Once created, the geometry can be modified to mimic airway obstruction caused by asthma or COPD. According to the way the geometry is created and stored, the diameter of each airway (i,j) in the seven generations of the asymmetric zone can be modified individually, and the diameter of all the airways belonging to the same generation of a given subtree can be modified together. According to the high sensitivity of the flow resistance in an airway to the airway diameter, as expressed by the Poiseuille law (∝ *D*^−1/4^), dividing the radius of the airway by a factor of 10 is equivalent to completely blocking the airway, with a transfer of flow to the adjacent airways.

### Airway resistance and ventilation distribution

For a given value of the (inspiratory or expiratory) flow rate of air in the trachea and a fixed geometry created by the “geom” function (after potentially constricting several airways), the objective is to calculate a steady-state distribution of the air flow in the lungs. More precisely, the goal is to calculate the flow rate in each airway (in the asymmetric zone and in the subtrees), as well as the pressure difference across the lungs due to viscous losses. This flow distribution is modeled following a classical electrical analogy, assigning a resistance to each airway and considering equivalences between flow rate and electrical current / pressure difference and electrical potential difference.

We assume a fully developed Poiseuille flow in the subtrees (their first generation excluded) and a homogeneous alveolar pressure (i.e., the same pressure at the end of each subtree). Therefore, the flow resistance in the entire subtree k (excluding its first generation) is

$$R_{k}^{\mathrm{sub}}=\sum_{i=2}^{l_{k}^{\mathrm{sub}}+7}\frac{1}{2^{i-1}}\frac{128\;\mu \;L_{k,i}^{\mathrm{sub}}}{\pi {\left(D_{k,i}^{\mathrm{sub}}\right)}^{4}}=\sum_{i=2}^{l_{k}^{\mathrm{sub}}}\frac{1}{2^{i-1}}\frac{128\;\mu \;\beta }{\pi h^{3}{\left(D_{k,1}^{\mathrm{sub}}\right)}^{3}}+\frac{1}{2^{l_{k}^{\mathrm{sub}}}}\sum_{s=1}^{7}\frac{1}{2^{s-1}}\frac{128\;\mu \;L_{a}}{\pi D_{a}^{4}}$$ Eq 9

Note that only the first equality in Eq 9 is valid if the geometry of the subtree k has been altered.

We can now consider the asymmetric zone. *Q*_1,1_ is the inspiratory or expiratory flow rate (i.e., the flow rate in the trachea) and *Q*_*i*,*j*_ is the flow rate in the airway (i,j) of the asymmetric zone. The average velocity of the air in generation (i,j) is $v_{i,j}=4Q_{i,j}/(\pi D_{i,j}^{2})$.

The Reynolds number of the flow in the airway (i,j) of the asymmetric zone is defined as

$${Re}_{i,j}=\frac{\rho v_{i,j}D_{i,j}}{\mu }=\frac{4{\rho Q}_{i,j}}{\mu \pi D_{i,j}}$$ Eq 10

with *ρ* and *μ* the density and viscosity of the air, respectively. They are evaluated at 37°C (for an air saturated with water) and their values are provided in Table 1.

It has been shown that the fact that the flow is not fully developed (i.e., different from a Poiseuille flow) in the first generations of the lungs contributes significantly to the total airway resistance [23]. Therefore, in the first seven generations of the lungs, the resistance to flow in the airway (i,j) is the product of the Poiseuille resistance and a factor *Z*_*i*,*j*_, to take into account the possible increase in wall gradients due to the not fully developed flow [23, 26]:

$$R_{i,j}=\frac{128\;\mu \;L_{i,j}}{\pi D_{i,j}^{4}}Z_{i,j}$$ Eq 11

with

Zi,j=Max[1,13Rei,jLi,jDi,j] Eq 12

with Max[a,b] the numerically largest of a and b. Please note that a factor 1/4 instead of 1/3 is used in [26]. However, the factor 1/3 is much closer to the one carefully derived by Pedley et al. [23], while the factor 1/4 is obtained in [26] by a simpler approach.

The peripheral resistance of the airway (i,j) in the asymmetric zone is defined as the resistance to flow exhibited by the entire part of the lungs downstream of the airway. It is written $R_{i,j}^{\mathrm{per}}$.

We have first: $R_{7,j}^{\mathrm{per}}=R_{k=j}^{\mathrm{sub}},\forall j=1,\ldots ,2^{6}$. Indeed, remember that the airways in generation 7 of the asymmetric zone are the first airways of the subtrees.

Then, we can also write, climbing in the lungs from generation 6 to 1 in the asymmetric zone:

$$R_{i,j}^{\mathrm{per}}={\left(\frac{1}{R_{i+1,2j-1}+R_{i+1,2j-1}^{\mathrm{per}}}+\frac{1}{R_{i+1,2j}+R_{i+1,2j}^{\mathrm{per}}}\right)}^{-1},\forall i=6,\ldots ,1;\;\forall j=1,\ldots ,2^{i-1}$$ Eq 13

To compute the flow distribution in the lungs for a given value of *Q*_1,1_, we now set up an iterative procedure. First, all the *Z*_*i*,*j*_ are put equal to 1. Then all the resistances ($R_{k}^{\mathrm{sub}}$ and *R*_*i*,*j*_) are evaluated, using Eq 9 and Eq 11, as well as the peripheral ones $R_{i,j}^{\mathrm{per}}$, using Eq 13.

The total airway resistance is then calculated as

$$R_{t}=R_{1,1}+R_{1,1}^{\mathrm{per}}$$

And the difference in pressure between the top of the trachea and the alveolar pressure due to viscous losses is

$$\Delta P=R_{t}Q_{1,1}$$ Eq 14

In this paper, Δ*P* is simply referred to as the alveolar pressure.

Consequently, the flowrates *Q*_*i*,*j*_ can be computed, going down from generation 2 to generation 7 in the asymmetric zone, using the following equations:

$$\begin{array}{c} Q_{i+1,2j-1}=Q_{i,j}\frac{R_{i+1,2j}+R_{i+1,2j}^{\mathrm{per}}}{R_{i+1,2j}+R_{i+1,2j}^{\mathrm{per}}+R_{i+1,2j-1}+R_{i+1,2j-1}^{\mathrm{per}}}\\ Q_{i+1,2j}=Q_{i,j}\frac{R_{i+1,2j-1}+R_{i+1,2j-1}^{\mathrm{per}}}{R_{i+1,2j}+R_{i+1,2j}^{\mathrm{per}}+R_{i+1,2j-1}+R_{i+1,2j-1}^{\mathrm{per}}} \end{array}\}\;\forall i=1,\ldots ,6;\;\forall j=1,\ldots ,2^{i-1}$$

Then, the Reynolds numbers are calculated with Eq 10, and the *Z*_*i*,*j*_ are reevaluated with Eq 12. The whole procedure is then repeated several times, until convergence is reached (measured by a relative variation of the total resistance *R*_*t*_ between two successive steps below 0.1%).

The average flow rate in the terminal airways of all subtrees (i.e., the average flow rate entering / leaving the alveolar region) is the flow rate in the trachea *Q*_1,1_ divided by the number of terminal airways *n*^ter^. Remember that each airway in generation 7 of the asymmetric zone is the first airway of a subtree. Thus, *Q*_7,*k*_ is the flow rate entering subtree k and $Q_{7,k}/2^{l_{k}-1}$ is the flow rate in its $2^{l_{k}-1}$ terminal airways. This allows to plot a histogram of the ratio between the flow rate in a terminal airway and the average flow rate in these airways, providing information about the heterogeneity of ventilation in the lungs.

The “flow” function in the Wolfram Mathematica file and in the Python files calculates the flow distribution (i.e., the different *Q*_*i*,*j*_, as well as the different *v*_*i*,*j*_), the total resistance to flow in the lungs *R*_*t*_ and the alveolar pressure Δ*P* (in cm of H_2_O) as functions of the flow rate in the trachea *Q*_1,1_ (in l/min) (inhalation or exhalation), on a geometry previously defined with the “geom” function (and possibly modified thereafter to mimic airway constriction). It also produces the above-mentioned histogram to describe the heterogeneity of the flow distribution in the acini.

The “dpQ” function in the Python and Wolfram Mathematica files generates a plot of the alveolar pressure Δ*P* (in cm of H_2_O) as a function of the flow rate in the trachea *Q*_1,1_ (in l/min), on a previously defined geometry.

### Fraction of exhaled nitric oxide (FeNO)

The objective here is to simulate the NO concentration at the top of the trachea at the end of a long exhalation at a constant flow rate and against a resistance (the so-called FeNO, expressed in ppb). In practice, it is measured at the mouth, but both can be considered equal, even if NO is also produced in the upper airways, because exhalation against a resistance means that the soft palate rises, blocking contact between the upper airways and the expiratory flow.

It has been shown that, even at the highest expiratory flow rate commonly used for FeNO measurement, a stationary state of the NO concentration profile in the lungs can be assumed to simulate FeNO [18, 26]. It is an important feature that allows repeatability of FeNO measurements.

Let us first recall that NO is physiologically produced in the epithelium of the airway walls [47]. It can then diffuse either toward the connective tissue, where it is bound to red blood cells in the capillaries, or toward the lumen of the airways through the ASL. We can thus introduce, in each airway of the lungs, a NO transfer flux, which expresses the amount of NO produced in the epithelium of the airway and transferred to its lumen, per unit of time and per unit of area of the ASL-lumen interface in the airway. It is written *J*_*i*,*j*_ for the airway (i,j) in the asymmetric zone and $J_{k,i}^{\mathrm{sub}}$ for the airways constituting generation i of the subtree k. In the strict sense, this transfer flux is influenced by the concentration of NO in the lumen (it decreases if this concentration increases). However, it has been shown to vanish for concentrations much higher than those reached in the lumen during a classical FeNO test. We can thus assume, without loss of generality, that this transfer flux does not depend on the concentration of NO in the lumen.

In healthy lungs, we assume that the NO transfer flux is homogeneous (i.e., $J_{i,j}(\forall i,j)=J_{k,i}^{\mathrm{sub}}(\forall k,i)=J_{\mathrm{NO}}$). A reference value for *J*_NO_ is given in Table 1 (in units such that the predicted FeNO expresses in ppb). Tsoukias and George report a value of 5 10^−11^ mol/s for the total production of NO in the epithelium of the airways of adult humans [37]. Dividing this value by the total surface area of the airway epithelium calculated when creating a lung geometry for a body mass of 75 kg (about 0.5 m^2^), and converting to units that yield FeNO in ppb, we obtain a value of 2.6 10^−3^ ppb.m^3^/(m^2^.s) for the NO transfer flux (at 1 atm and 37°C). However, this value should only be considered as a good order of magnitude, since the value given by Tsoukias and George for the total production rate of NO is the result of fitting the parameters of a model, and since part of the NO produced in the epithelium diffuses toward the lamina propria where it is consumed by blood. Therefore, our reference value given in Table 1 is rounded to 2 10^−3^ ppb.m^3^/(m^2^.s), which, as shown in the Results section, gives a good agreement between our model and the data of Silkoff et al. [47].

When the geometry of healthy lungs is created with the “geom” function, the NO transfer flux in each airway of the lungs (asymmetric zone and subtrees) is assigned the value of *J*_NO_ given in Table 1. This can be modified later to mimic the increased NO production in the epithelium of airways associated with local or global inflammation in asthma. According to the way the geometry is created and stored, the NO transfer flux in each airway (i,j) in the seven generations of the asymmetric zone can be modified individually, and the NO transfer fluxes in all airways belonging to the same generation of a given subtree can be modified together.

First, using the “flow” function on a previously defined geometry, a steady state flow distribution during exhalation at a given flow rate is calculated (i.e., the different *Q*_*i*,*j*_ and *v*_*i*,*j*_ are calculated for a given value of *Q*_1,1_). Then, as a second step, we must establish equations to describe the transport of NO in the lumen of the airways during this exhalation.

Two mechanisms of NO transport coexist in the airway lumen during a FeNO test. First, the expiratory flow generates a convective transport of NO toward the top of the trachea. Second, the lowest NO concentration is actually achieved in the lumen of the alveoli. In fact, due to the high affinity of blood for NO, the equilibrium concentration of NO in the alveolar lumen is approximately 2 ppb. Consequently, there is an axial diffusion flux of NO toward the alveoli where it is consumed. This is commonly referred to as "back-diffusion" [25].

In generation (i,j) of the asymmetric zone, the Péclet number Pe_*i*,*j*_ compares a characteristic time of NO transport by convection (*L*_*i*,*j*_/*v*_*i*,*j*_) with a characteristic time of axial NO transport by diffusion ($L_{i,j}^{2}/D_{\mathrm{NO}}$), with *D*_NO_ the diffusion coefficient of NO in air (whose value at 37°C is provided in Table 1):

Pei,j=Li,j2DNOLi,jvi,j=Li,jvi,jDNO

A Péclet number can also be defined to describe the competition between convection and diffusion in generation i of the subtree k:

$${\mathrm{Pe}}_{k,i}^{\mathrm{sub}}=\frac{L_{k,i}^{\mathrm{sub}}v_{k,i}^{\mathrm{sub}}}{D_{\mathrm{NO}}}$$

with $v_{k,i}^{\mathrm{sub}}$ the average velocity in an airway in generation i of the subtree k:

$$v_{k,i}^{\mathrm{sub}}=\frac{Q_{7,k}}{2^{i-1}}\frac{4}{\pi {\left(D_{k,i}^{\mathrm{sub}}\right)}^{2}}$$

Remember that each airway in generation 7 of the asymmetric zone is the beginning of a subtree. Therefore, *Q*_7,*k*_ is the flow rate entering the subtree k and thus *Q*_7,*k*_/2^*i*−1^ is the flow rate in its generation i.

A Péclet number much greater than 1 means that convection dominates diffusion (and thus that net NO transport is toward the trachea during exhalation), whereas a Péclet number much less than 1 means that diffusion dominates convection (and thus that the net NO transport is toward the alveoli due to the low NO concentration there).

The Péclet number decreases as one moves along the lungs toward the alveolar region. In the asymmetric zone, it can be calculated that, even at the lowest expiratory flow rate commonly used for FeNO measurement, the values of Pe_*i*,*j*_ are greater than 10, meaning that convection is the dominant mechanism of NO transport in the asymmetric zone during a FeNO test. In the alveolar part of the subtree, the Peclet number can reach values much less than 1, which means that diffusion is the main mechanism of NO transport in the distal region of lungs during a FeNO test.

#### Subtrees

The NO transport in the subtrees during a FeNO test is modeled by a classical 1D approach, as in many previous works [18, 22, 25, 26].

In each generation i of a subtree k, we introduce a *z* axis to measure the axial position in the airways of this generation. *z* is equal to zero at the distal end (i.e., farthest from the trachea) of the airways and equal to $L_{k,i}^{\mathrm{sub}}$ at their proximal end. We write $C_{k,i}^{\mathrm{sub}}(z)$ the stationary state NO concentration during a FeNO test, at position *z* in the lumen of the airways in generation i of the subtree k.

We can now establish a mass balance equation on a slice [*z*,*z*+Δ*z*] of the lumen of an airway in generation i of the subtree k, to describe NO transport at stationary state during a FeNO test:

$$\frac{\pi }{4}{\left(D_{k,i}^{\mathrm{sub}}\right)}^{2}\left(v_{k,i}^{\mathrm{sub}}C_{k,i}^{\mathrm{sub}}(z)-D_{\mathrm{NO}}{\frac{dC_{k,i}^{\mathrm{sub}}}{dz}|}_{z}\right)+({1-\theta }_{k,i}^{\mathrm{sub}})\pi D_{k,i}^{\mathrm{sub}}\Delta zJ_{k,i}^{\mathrm{sub}}=\frac{\pi }{4}{\left(D_{k,i}^{\mathrm{sub}}\right)}^{2}\left(v_{k,i}^{\mathrm{sub}}C_{k,i}^{\mathrm{sub}}(z+\Delta z)-D_{\mathrm{NO}}{\frac{dC_{k,i}^{\mathrm{sub}}}{dz}|}_{z+\Delta z}\right)+\gamma_{k,i}^{\mathrm{sub}}\omega_{k,i}^{\mathrm{sub}}\pi D_{k,i}^{\mathrm{sub}}\Delta zk_{\mathrm{NO}}(C_{k,i}^{\mathrm{sub}}(z)-C_{\mathrm{eq}})$$ Eq 15

The first term on the left side of this equation is the NO flux entering the slice [*z*,*z*+Δ*z*] by convection / diffusion in the lumen. The first term on the right side of this equation is the NO flux leaving this slice by convection / diffusion in the lumen. The second term on the left side is the amount of NO produced in the epithelium of the airway wall and transferred to the lumen per unit of time in the slice $[z,z+\Delta z].\;\theta_{k,i}^{\mathrm{sub}}$ is a coefficient that evaluates the fraction of the lateral wall of the airway that is occupied by alveoli (with a maximum value of 1, usually reached in the third generation of the alveolar part of the subtrees). It is calculated assuming that each alveolus blocks a surface on the airway wall with an area equal to the cross-section $\pi d_{\mathrm{alv}}^{2}/4$ of the alveolus. Therefore, we have

$$\theta_{k,i}^{\mathrm{sub}}=\left\{ \begin{array}{c} 0,\forall i=1,\ldots ,l_{k}^{\mathrm{sub}}\\ Min[\frac{n_{\mathrm{alv},s}\frac{\pi d_{\mathrm{alv}}^{2}}{4}}{\pi D_{k,i}^{\mathrm{sub}}L_{k,i}^{\mathrm{sub}}},1],for\;i=l_{k}^{\mathrm{sub}}+s(\forall s=1,\ldots ,7) \end{array}\right.$$

We multiply the second term on the left side of Eq 15 by ${1-\theta }_{k,i}^{\mathrm{sub}}$ to express the fact that the alveoli reduce (and eventually completely cancel out) the presence of bronchial-type epithelium within the alveolar part of the subtrees.

The second term on the right side of Eq 15 gives the amount of NO consumed per unit of time by the alveoli in the slice [*z*,*z*+Δ*z*] (i.e., consumed by the blood in the capillaries surrounding these alveoli). *C*_eq_ is the NO concentration in the alveoli at equilibrium with the blood in the capillaries, whose value is given in Table 1, and *k*_NO_ is the mass transfer coefficient of NO across the alveolar membrane, which is evaluated as the ratio of the diffusion coefficient of NO through the tissue (3 10^−9^ m^2^/s) to the thickness of the alveolar membrane (10^−6^ m). $\pi D_{k,i}^{\mathrm{sub}}\Delta z$ is the lateral surface area of an airway in generation i of the subtree k and $\omega_{k,i}^{\mathrm{sub}}$ is a coefficient expressing the ratio of the surface area of the alveoli (hemispheres with diameter *d*_alv_) in the airway to the lateral surface area of the airway:

$$\omega_{k,i}^{\mathrm{sub}}=\left\{ \begin{array}{c} 0,\forall i=1,\ldots ,l_{k}^{\mathrm{sub}}\\ \frac{n_{\mathrm{alv},s}\frac{\pi d_{\mathrm{alv}}^{2}}{2}}{\pi D_{i,k}^{\mathrm{sub}}L_{i,k}^{\mathrm{sub}}},for\;i=l_{k}^{\mathrm{sub}}+s(\forall s=1,\ldots ,7) \end{array}\right.$$

Finally, $\gamma_{k,i}^{\mathrm{sub}}$ in the second term on the right side of the balance equation is a coefficient describing the state of perfusion of the alveoli in the airway (if there are any). $\gamma_{k,i}^{\mathrm{sub}}=1$ means maximum perfusion, while $\gamma_{k,i}^{\mathrm{sub}}=0$ means no perfusion. When the geometry of healthy lungs is created (in the “geom” function), the values of $\gamma_{k,i}^{\mathrm{sub}}$ are assigned based on the classical 3-zone model [38]: no perfusion in the upper part of the lungs, maximal perfusion in the lower part and a transition zone between the two. We express this as:

$$\gamma_{k,i}^{\mathrm{sub}}=\left\{ \begin{array}{c} 0,\forall k=1,\ldots ,21\;and\;\forall i=1,\ldots ,l_{k}^{\mathrm{sub}}+7\\ \frac{k-22}{43-22},\forall k=22,\ldots ,43\;and\;\forall i=1,\ldots ,l_{k}^{\mathrm{sub}}+7\\ 1,\forall k=44,\ldots ,64\;and\;\forall i=1,\ldots ,l_{k}^{\mathrm{sub}}+7 \end{array}\right.$$ Eq 16

Each $\gamma_{k,i}^{\mathrm{sub}}$ can be modified individually, to mimic a perfusion change.

Eq 15 can be simplified into the following stationary convection-diffusion equation to describe the NO transport in an airway in generation i of the subtree k during a FeNO test:

$$v_{k,i}^{\mathrm{sub}}\frac{dC_{k,i}^{\mathrm{sub}}}{dz}=D_{\mathrm{NO}}\frac{d^{2}C_{k,i}^{\mathrm{sub}}}{{dz}^{2}}+\frac{4}{D_{k,i}^{\mathrm{sub}}}({1-\theta }_{k,i}^{\mathrm{sub}})J_{k,i}^{\mathrm{sub}}-{\frac{4}{D_{k,i}^{\mathrm{sub}}}\gamma }_{k,i}^{\mathrm{sub}}\omega_{k,i}^{\mathrm{sub}}k_{\mathrm{NO}}(C_{k,i}^{\mathrm{sub}}(z)-C_{\mathrm{eq}})$$ Eq 17

Please note that $v_{k,i}^{\mathrm{sub}}$ is set to zero in the last x generations of the subtrees, to model them as a deflating balloon. The results show no sensitivity to x if it is between 1 and 3.

This transport equation can be written for each generation of the subtree k. It is completed by the following boundary conditions.

Convection is the dominant mechanism of mass transport in the asymmetric zone, and thus also in the first generation of the subtrees. Therefore, we can assume a zero diffusive flux at the proximal end of the first generation of the subtrees:

$${\frac{dC_{k,1}^{\mathrm{sub}}}{dz}|}_{z=L_{k,1}^{\mathrm{sub}}}=0$$ Eq 18

The continuity of the concentration between successive generations writes

$$C_{k,i}^{\mathrm{sub}}(0)=C_{k,i+1}^{\mathrm{sub}}(L_{k,i+1}^{\mathrm{sub}}),\forall i=1,\ldots ,l_{k}^{\mathrm{sub}}+6$$ Eq 19

The continuity of the flux between successive generations writes

$${\frac{\pi }{4}(D_{k,i}^{\mathrm{sub}})}^{2}\left(v_{k,i}^{\mathrm{sub}}C_{k,i}^{\mathrm{sub}}(0)-D_{\mathrm{NO}}{\frac{dC_{k,i}^{\mathrm{sub}}}{dz}|}_{z=0}\right)={2\frac{\pi }{4}(D_{k,i+1}^{\mathrm{sub}})}^{2}\left({v_{k,i+1}^{\mathrm{sub}}C_{k,i+1}^{\mathrm{sub}}(L_{k,i+1}^{\mathrm{sub}})-D_{\mathrm{NO}}\frac{dC_{k,i+1}^{\mathrm{sub}}}{dz}|}_{z=L_{k,i+1}^{\mathrm{sub}}}\right)$$ Eq 20

Finally, a zero diffusive flux is imposed at the distal end of the last generation of subtrees:

$${\frac{dC_{k,l_{k}^{\mathrm{sub}}+7}^{\mathrm{sub}}}{dz}|}_{z=0}=0$$ Eq 21

Solving Eq 17–Eq 21 for a given subtree allows to calculate the NO concentration at the beginning of the subtree (i.e., in the flow leaving the subtree): $C_{k,1}^{\mathrm{sub}}(L_{k,1}^{\mathrm{sub}})$. This is easily done in Wolfram Mathematica with the built-in NDSolve function (and in Python with the solve_bvp function). Note that in the code we perform a rescaling of *z* from 0 to 1 in each generation to simplify the implementation.

#### Asymmetric zone

Let $C_{i,j}^{p}$ and $C_{i,j}^{d}$ be the NO concentrations, at stationary state during a FeNO test, at the proximal (i.e., the closest to the trachea) and the distal ends of the airway (i,j).

First, we have $C_{7,j}^{p}=C_{k=j,1}^{\mathrm{sub}}(z=L_{k,1}^{\mathrm{sub}}),\forall j=1,\ldots ,2^{6}$

Then, as convection is the dominant mechanism of NO transport in the lumen of airways in the asymmetric zone, we can climb the tree:

$$\begin{array}{c} C_{i,j}^{d}=\frac{Q_{i+1,2j}C_{i+1,2j}^{p}+Q_{i+1,2j-1}C_{i+1,2j-1}^{p}}{Q_{i,j}}\\ C_{i,j}^{p}=\frac{Q_{i,j}C_{i,j}^{d}+\pi D_{i,j}L_{i,j}J_{i,j}}{Q_{i,j}} \end{array}\}\;\forall i=6,\ldots ,1;\;\forall j=1,\ldots ,2^{i-1}$$

And thus, we obtain FeNO = $C_{1,1}^{p}$.

The “FeNO” function in the Python and Wolfram Mathematica files calculates FeNO (in ppb) for a given value of the expiratory flow rate *Q*_1,1_ (in l/min), on a previously defined geometry. It starts by calculating the exhalation flow distribution by using the “flow” function. After the “FeNO” function has been used, the “profileNO” function can be called to give a graphical representation of the NO concentration profiles in the lungs. The “relfenodeb” function generates a plot of the FeNO (in ppb) as a function of the expiratory flow rate *Q*_1,1_ (in l/min), on a previously defined geometry. It also shows the well-known experimental data of Silkoff et al. on this plot [47], to allow comparison with a healthy subject.

### Diffusing capacity for nitric oxide

A DLNO test starts with the rapid inhalation of a given volume of gas *V*^insp^ (about 4 l), in a time *t*^insp^ (less than 1 second). It thus defines an inspiratory flow rate ${Q^{\mathrm{insp}}=Q}_{1,1}=V^{\mathrm{insp}}/t^{\mathrm{insp}}$. The inhaled gas contains NO, at a concentration of approximately 40 ppm. After inhalation, there is a breath-hold of a few seconds (the duration of which is written *t*^bh^), during which the inhaled NO diffuses into the lumen of the lungs and is consumed in the alveoli. Finally, a rapid exhalation is performed and the amount of NO in the exhaled air is measured [39]. Another inert (i.e., not consumed) species, typically helium, is also introduced into the inhaled air. The amount of this inert compound recovered in the exhaled air allows determination of the amount of NO remaining in the lungs at the end of the exhalation. Consequently, it is possible to calculate the fraction of inhaled NO that is consumed in the alveoli.

The concentration of NO in the inhaled volume is about 40 ppm. This is much higher than the (patho)physiological concentration of NO in the lumen of lungs (even under conditions of severe inflammation). Therefore, interactions with the airway epithelium, where (patho)physiological NO is produced, can be neglected and the equilibrium concentration in the alveoli can be considered as zero. Consequently, the fraction of inhaled NO consumed in the alveoli is independent of the NO concentration in the inhaled gas. We can therefore simply simulate a DLNO test by imposing a dimensionless NO concentration in the inhaled gas equal to 1.

We assume that the first six generations of the asymmetric zone are rigid (no dimensional change during inhalation and exhalation), which makes sense due to the presence of cartilaginous tissue around the airways in these generations. The subtrees inflate during inhalation and deflate during exhalation. The way the inhaled volume is distributed among the different subtrees is obtained by calculating, using the “flow” function, the different *Q*_*i*,*j*_ resulting from the flow rate *Q*_1,1_ = *V*^insp^/*t*^insp^, on the previously created geometry.

As a reminder, before inhalation, the volume of the subtree k is (see Eq 5)

$$V_{k}^{\mathrm{sub}}=\sum_{i=1}^{l_{k}^{\mathrm{sub}}+7}2^{i-1}\frac{\pi }{4}{\left({\bar{D}}_{k,i}^{\mathrm{sub}}\right)}^{2}L_{k,i}^{\mathrm{sub}}$$

After inhalation, the volume of the subtree k is increased by an amount *Q*_7,*k*_*t*^insp^. We assume that the increased volume of this subtree k is the result of an increase in the diameter of its airways $D_{k,i}^{\mathrm{sub}}$ (with constant length) and alveoli *d*_alv_. The updated values of these diameters after inhalation, written $D_{k,i}^{\mathrm{sub},*}$ and $d_{\mathrm{alv},k}^{*}$ respectively, are calculated assuming the same volumetric expansion ratio of all structures (with fixed airway length):

$$\begin{array}{c} D_{k,i}^{\mathrm{sub},*}=\sqrt{\varepsilon_{k}}D_{k,i}^{\mathrm{sub}}\\ d_{alv,k}^{*}=\sqrt[{3}]{\varepsilon_{k}}d_{\mathrm{alv}} \end{array}\},\forall i=1,\ldots ,l_{k}^{\mathrm{sub}}+7$$

with

$$\varepsilon_{k}=1+\frac{Q_{7,k}t^{\mathrm{insp}}}{V_{k}^{\mathrm{sub}}}$$

the ratio of the increased volume of the subtree k after inhalation to its initial volume.

We can also update the value of the “total volume” diameter ${\bar{D}}_{k,i}^{\mathrm{sub}}$ (see Eq 3):

$${\bar{D}}_{k,i}^{\mathrm{sub},*}=D_{k,i}^{\mathrm{sub},*}\sqrt{1+n_{\mathrm{alv},s}\frac{{\left(d_{alv,k}^{*}\right)}^{3}}{3L_{k,i}^{\mathrm{sub}}{\left(D_{k,i}^{\mathrm{sub},*}\right)}^{2}}}=\sqrt{\varepsilon_{k}}{\bar{D}}_{k,i}^{\mathrm{sub}}$$

The volume of the subtree k after inhalation is

$$V_{k}^{\mathrm{sub},*}=\sum_{i=1}^{l_{k}^{\mathrm{sub}}+7}2^{i-1}\frac{\pi }{4}{\left({\bar{D}}_{k,i}^{\mathrm{sub},*}\right)}^{2}L_{k,i}^{\mathrm{sub}}$$

As already written (see Eq 4), the volume of the first 6 generations is

$$V_{1}=\sum_{i=1}^{6}\sum_{j=1}^{2^{i-1}}\frac{\pi }{4}D_{i,j}^{2}L_{i,j}$$

*V*_1_ is about 60 ml for a body mass of 70 kg, which is negligible in front of *V*^insp^ for a DLNO test (about 4 l). So we can forget that the inhaled air also occupies the first 6 generations of the lungs and assume that *Q*_7,*k*_*t*^insp^ is also the volume of inhaled air entering the subtree k.

It is now important to define the proper initial conditions for the breath-hold phase. First, we assume that, due to the large Péclet numbers generated by the inhalation of a large volume in a short time, no diffusion of NO occurs during inhalation (i.e., the inhaled NO is transported purely by convection during inhalation). Then, we define *f*_*k*,*i*_ as the ratio of the cumulated volume of generations 1 to i of the subtree k to the total volume of the subtree:

$$f_{k,i}=\frac{\sum_{j=1}^{i}2^{j-1}\frac{\pi }{4}{\left({\bar{D}}_{k,j}^{\mathrm{sub},*}\right)}^{2}L_{k,j}^{\mathrm{sub}}}{V_{k}^{\mathrm{sub},*}}$$

Let *λ*_*K*_ be the index of the generation such that the inhaled volume in the subtree k fully occupies generations 1 to *λ*_*K*_ of the subtree and partially occupies generation *λ*_*K*_+1.*λ*_*K*_ is thus such that

$$\frac{Q_{7,k}t^{\mathrm{insp}}}{V_{k}^{\mathrm{sub},*}}>f_{k,\lambda_{k}}\;and\;\frac{Q_{7,k}t^{\mathrm{insp}}}{V_{k}^{\mathrm{sub},*}}<f_{k,\lambda_{k}+1}$$

Generation *λ*_*K*_+1 of the subtree k is thus partially occupied by inhaled air. The ratio of the volume of this generation occupied by inhaled air to the total volume of this generation is

$$\varphi_{k}=\frac{\frac{Q_{7,k}t^{\mathrm{insp}}}{V_{k}^{\mathrm{sub},*}}-f_{k,\lambda_{k}}}{f_{k,\lambda_{k}+1}-f_{k,\lambda_{k}}}$$

In each generation i of the subtree k, we introduce again a *z* axis to measure the axial position in the airways of that generation. *z* is equal to zero at the distal end (i.e., the farthest from the trachea) of the airways and equal to $L_{k,i}^{\mathrm{sub}}$ at their proximal end. Then, $C_{k,i}^{\mathrm{sub}}(z,t)$ is the NO concentration at position *z* and at time *t* (with *t* = 0 at the beginning of the breath-hold phase of the DLNO test) in the lumen of the airways in generation i of the subtree k.

Finally, we define, as initial conditions of the breath-hold phase:

$$C_{k,i}^{\mathrm{sub}}(z,0)=\left\{ \begin{array}{c} 1,if\;i\leq \lambda_{k}\\ 1,if\;i=\lambda_{k}+1\;\mathrm{and}\;\frac{z}{L_{k,i}^{\mathrm{sub}}}<\varphi_{k}\\ 0,if\;i=\lambda_{k}+1\;\mathrm{and}\;\frac{z}{L_{k,i}^{\mathrm{sub}}}>\varphi_{k}\\ 0,if\;{i>\lambda }_{k}+1 \end{array}\right.$$ Eq 22

Now, we can write a balance equation on a slice [*z*,*z*+Δ*z*] of the lumen of an airway in generation i of the subtree k, to simulate diffusion and consumption by the alveoli during the breath-hold phase of a DLNO test:

$$\frac{\pi }{4}{\left({\bar{D}}_{k,i}^{\mathrm{sub},*}\right)}^{2}\Delta z\frac{\partial C_{k,i}^{\mathrm{sub}}}{\partial t}=-\frac{\pi }{4}{\left(D_{k,i}^{\mathrm{sub},*}\right)}^{2}D_{\mathrm{NO}}{\frac{\partial C_{k,i}^{\mathrm{sub}}}{\partial z}|}_{z}+\frac{\pi }{4}{\left(D_{k,i}^{\mathrm{sub},*}\right)}^{2}D_{\mathrm{NO}}{\frac{\partial C_{k,i}^{\mathrm{sub}}}{\partial z}|}_{z+\Delta z}-\gamma_{k,i}^{\mathrm{sub}}\frac{\Delta z}{L_{k,i}}{\psi_{k,i}\frac{\pi {\left(d_{alv,k}^{*}\right)}^{2}}{2}k}_{\mathrm{NO}}C_{k,i}^{\mathrm{sub}}(z,t)$$

with *ψ*_*k*,*i*_ = 0 if $i\leq l_{k}^{sub}$ and *ψ*_*k*,*i*_ = *n*_alv,*s*_ for $i=l_{k}^{\mathrm{sub}}+s(\forall s=1,\ldots ,7)$

This equation can be rearranged into

$$\frac{\partial C_{k,i}^{\mathrm{sub}}}{\partial t}={\left(\frac{D_{k,i}^{\mathrm{sub}}}{{\bar{D}}_{k,i}^{\mathrm{sub}}}\right)}^{2}D_{\mathrm{NO}}\frac{\partial^{2}C_{k,i}^{\mathrm{sub}}}{\partial z^{2}}-\gamma_{k,i}^{\mathrm{sub}}\frac{2}{L_{k,i}}\frac{\psi_{k,i}}{\sqrt[{3}]{\varepsilon_{k}}}{\left(\frac{d_{\mathrm{alv}}}{{\bar{D}}_{k,i}^{\mathrm{sub}}}\right)}^{2}k_{\mathrm{NO}}C_{k,i}^{\mathrm{sub}}(z,t)$$ Eq 23

This transport equation must be completed by boundary conditions.

During the breath-hold phase, the results show that the diffusion of NO toward the alveoli, where it is consumed, does not affect the concentration in the first generation of subtrees. This means that this concentration remains around 1 during the breath-hold phase and the subtrees do not interact during this phase. Therefore, we can assume a zero diffusive flux at the proximal end of the first generation of the subtrees:

$${\frac{\partial C_{k,1}^{\mathrm{sub}}}{\partial z}|}_{z=L_{k,1}^{\mathrm{sub}}}=0$$ Eq 24

The continuity of the concentration between successive generations writes

$$C_{k,i}^{\mathrm{sub}}(0)=C_{k,i+1}^{\mathrm{sub}}(L_{k,i+1}^{\mathrm{sub}}),\forall i=1,\ldots ,l_{k}^{\mathrm{sub}}+6$$ Eq 25

While the continuity of the flux between successive generations writes

$${\frac{\pi }{4}(D_{k,i}^{\mathrm{sub},*})}^{2}D_{\mathrm{NO}}{\frac{\partial C_{k,i}^{\mathrm{sub}}}{\partial z}|}_{z=0}={2\frac{\pi }{4}(D_{k,i+1}^{\mathrm{sub},*})}^{2}{D_{\mathrm{NO}}\frac{\partial C_{k,i+1}^{\mathrm{sub}}}{\partial z}|}_{z=L_{k,i+1}^{\mathrm{sub}}}$$ Eq 26

Finally, a zero diffusive flux is imposed at the distal end of the last generation of the subtrees:

$${\frac{\partial C_{k,l_{k}^{\mathrm{sub}}+7}^{\mathrm{sub}}}{\partial z}|}_{z=0}=0$$ Eq 27

In the Python and Wolfram Mathematica files, we implement a classical numerical solution of Eq 22–Eq 27, by spatial discretization of the diffusion term, thus transforming the partial differential equations into ordinary differential equations, and then using built-in functions to solve these ODE’s. Note that, in the code, we perform a rescaling of time *t* (with *t*^bh^) and axial position *z* (with $L_{k,i}^{\mathrm{sub}}$) to facilitate the implementation.

To simulate the rapid exhalation, we again assume that no diffusion of NO takes place during this exhalation (i.e., the remaining NO is transported only by convection during the exhalation). Consequently, the exhalation can be treated as the inhalation: at the end of the breath-hold, the NO present in the zone of the lungs initially occupied by the inhaled air (with thus $C_{k,i}^{\mathrm{sub}}(z,0)=1$) will leave the lungs during exhalation, while the remaining NO will move by convection toward the trachea, but will remain in the lungs.

After numerical solution of the equations describing the breath-hold phase, the amount of NO that is exhaled after the breath-hold (*A*^exp^) is calculated by integrating, for each subtree k, the concentration profile (at *t* = *t*^bh^) in the part of the subtree that was filed with fresh air at the end of the inhalation phase, and then summing on all the subtrees:

$$A^{\exp}=\sum_{k=1}^{2^{6}}\left((\sum_{i=1}^{\lambda_{k}}2^{i-1}\int_{0}^{L_{k,i}^{\mathrm{sub}}}C_{k,i}^{\mathrm{sub}}(z,t^{\mathrm{bh}}){\frac{\pi }{4}({\bar{D}}_{k,i}^{\mathrm{sub},*})}^{2}dz)+2^{\lambda_{k}}\int_{0}^{{\varphi_{k}L}_{k,\lambda_{k}+1}^{\mathrm{sub}}}C_{k,\lambda_{k}+1}^{\mathrm{sub}}(z,t^{\mathrm{bh}}){\frac{\pi }{4}({\bar{D}}_{k,\lambda_{k}+1}^{\mathrm{sub},*})}^{2}dz\right)$$

The average concentration of NO in the exhaled air is *ξ*^exp^ = *A*^exp^/*V*^insp^. It is also the fraction of the inhaled NO that is recovered in the exhaled air (as the NO concentration in the inhaled air is set to 1 in the model). Consequently, according to [41], DLNO is calculated as

$$\mathrm{DLNO}=\frac{V^{\mathrm{insp}}\mathrm{Log}(\frac{1}{\xi^{\exp}})}{t^{bh}(P_{\mathrm{atm}}-P_{\mathrm{sat},H_{2}O})}$$ Eq 28

with *P*_atm_ the atmospheric pressure and $P_{\mathrm{sat},H_{2}O}$ the saturation pressure of water at body temperature.

The amount of NO remaining in the lungs after the exhalation (*A*^rem^) is

$$A^{\mathrm{rem}}=\sum_{k=1}^{2^{6}}\left(\sum_{i=1}^{l_{k}^{\mathrm{sub}}+7}2^{i-1}\int_{0}^{L_{k,i}^{\mathrm{sub}}}C_{k,i}^{\mathrm{sub}}(z,t^{\mathrm{bh}}){\frac{\pi }{4}({\bar{D}}_{k,i}^{\mathrm{sub},*})}^{2}dz\right)-A^{\exp}$$

*ξ*^rem^ = *A*^rem^/(*Q*^insp^*t*^insp^) is the fraction of the inhaled NO which remains in the lungs at the end of the breath-hold phase (i.e., which is neither consumed nor exhaled).

Finally, the fraction of the inhaled NO which is consumed by the alveoli during breath-hold is *ξ*^con^ = 1−(*A*^exp^+*A*^rem^)/(*Q*^insp^*t*^insp^).

In the Python and Wolfram Mathematica files, the “DLNO” function calculates, on a previously defined geometry, the values of *A*^exp^ and DLNO as functions of *V*^insp^,*t*^insp^, and *t*^*bh*^. The “profileDLNO” function generates a plot of the concentration field of NO in the lungs at the end of a DLNO test.

### Pathological lungs

Once created, healthy lungs can be modified within the computational framework.

To simulate bronchoconstriction, we have implemented a function that allows to reduce by a factor of 10 (blocking all the flow) the diameter of the single airway (i,j) in the asymmetric zone (*D*_*i*,*j*_), or the diameter of all the airways in generation i of the subtree k ($D_{k,i}^{\mathrm{sub}}$). Note that in the latter case, the “total volume” diameter ${\bar{D}}_{k,i}^{\mathrm{sub}}$ must also be modified, according to Eq 3.

To simulate inflammation, we have implemented a function that allows to increase by a factor x the NO transfer flux in the single airway (i,j) in the asymmetric zone *J*_*i*,*j*_, or the NO transfer flux in all the airways in generation i of the subtree k $J_{k,i}^{\mathrm{sub}}$. Note that this NO transfer flux expresses the amount of NO produced in the epithelium of an airway and transferred to its lumen.

To simulate a change in perfusion, we have implemented a function that allows to completely or partially remove the perfusion in a subtree. In other words, this function allows to set to zero the perfusion coefficient $\gamma_{k,i}^{\mathrm{sub}}$, defined in Eq 16, for a given value of k, in the whole subtree ($\forall i=1,\ldots ,l_{k}^{\mathrm{sub}}+7$) or a part of it (selected values of i).

As illustrated in the Results section, these different functions can be combined / used multiple times to generate different pathological situations in the lungs, allowing to analyze their impact on airway resistance, FeNO and DLNO. Note that the above functions for modifying the lungs are only implemented in the Python file, since the latter was used to generate all the figures in the Results section.

## Supporting information

S1 File Python file of the computational framework. (ZIP)

S2 File Wolfram Mathematica file of the computational framework. (ZIP)
