# Supplementary material for: A new computational framework for simulating airway resistance, fraction of exhaled nitric oxide, and diffusing capacity for nitric oxide
Source: PLoS One. 2025 Jan 30;20(1):e0311667. doi: 10.1371/journal.pone.0311667 (PMC11781630; doi:10.1371/journal.pone.0311667)
Supplement: Attachment — Submitted filename: Answers to the reviewers.pdf [file pone.0311667.s003.pdf]

Dear Editor, Dear Reviewer,

Here are our responses to the editorial team's comments and the reviewer's comments. We believe we have responded to all of them.

I remain at your disposal for any further information.

Best regards,

Benoit Haut

Answer to editorial team:

The left side of Figure 1 was drawn from scratch by one of the authors (Cyril Karamaoun). The right side is already licensed under the Creative Commons Attribution 2.5, and this is properly referenced in the figure legend: "*Image by Patrick J. Lynch, medical illustrator / Wikimedia Commons. License: Creative Commons Attribution 2.5 License 2006. Bronchial anatomy detail of alveoli and pulmonary circulation*". (see: https://commons.wikimedia.org/wiki/File:Bronchial_anatomy.jpg). This image can be used freely.

Figure 2 was drawn entirely from scratch by Clément Rigaut.

The document format should meet all requirements.

Three references have been added to this revised version at the request of the reviewers. All other references are to previously published articles.

Answers to the reviewers:

Major comments:

1. Figures 4, 6, 7, 8, 10, and 11 all have used three different values of body mass. Please use a consistent value or report the curves for all the values.

We have followed your recommendation, choosing a mass of 75 kg for all these figures (average for adults in Belgium), except for figure 7. For this figure, we prefer to retain a mass of 80 kg (average for adult men only), as the results are compared with Silkoff's data, obtained mainly for men (8 out of 10 subjects). When a figure has been modified, the corresponding text has been adapted if necessary.

2. Figure 8 – Could you please elaborate as to why the NO concentration is discontinuous at the branching points?

Yes, of course. We have added the following comment in the text (page 17):

The discontinuities observed in the NO concentration profiles occur at the junctions between airways in the asymmetric zone. At this point, two airflows meet during exhalation, and these two flows have a priori different NO concentrations because they have different flow rates and have traveled a different path from the alveolar sacs (i.e., they have received different amounts of NO from the bronchial epithelium). Instantaneous mixing at the junction causes a jump in concentration, which becomes equal to the average of the concentrations in the two flows, weighted by their flow rates.

3. While the study results indicate that the model can capture the average behavior for a given body weight, could the authors discuss how the model could be used to simulate outliers in a subject-specific manner?

Currently, the user of the computational framework only needs to specify the subject's mass, and the geometry of the lungs is automatically generated by the code. Thus, a “standard” geometry is generated, which does not allow for the consideration of a patient that is an outlier in term of lung morphology. However, a simple extension of the code could be to allow the user to modify the parameters that define the geometry of the first 7 generations (asymmetric zone), to simulate the different tests for a specific patient.

We have added a comment about this in a discussion about future improvements (page 24).

4. While the ventilation tests offer confounded results, dynamic/functional imaging appears to be a method to determine regional ventilation and gas concentration. Could the authors discuss the possibility of estimating regional ventilation/NO concentration that matches the observed scans? In addition, medical Imaging has emerged as a significant diagnostic tool In assessing lung health/function. The need to create patient-specific lung geometry and airways from medical images will be relevant to this study. Please add a brief discussion in the Introduction and Discussion and cite relevant works such as:
https://doi.org/10.1117/12.2653648
https://doi.org/10.1111/j.1440-1843.2011.02117.x.

Also, the synthetic generation of airways (https://doi.org/10.1117/12.3006973) is relevant to this study.

Thanks for this valuable comment. We have added these three citations in the introduction / conclusion, thanks for the info. In particular, they are introduced in support of the discussion on future developments of the model (see below) and on the relevance of medical imaging as a diagnostic tool (introduction).

5. Please include a limitation subsection in section 3, discussing potential limitations and avenues for improvement.

Thanks for the advice. We have added the following discussion about some limitations and future improvements of the computational framework. However, instead of presenting it in Section 3, we think this discussion is more appropriate in the conclusion,

since the limitations naturally call for future developments, which are well presented in a conclusion.

Currently, the user of the computational framework only needs to specify the subject's mass, and the geometry of the lung is automatically generated by the code. Thus, a "standard" geometry is generated, which does not allow for the consideration of a patient that is an outlier in term of lung morphology. However, a simple extension of the code could be to allow the user to modify the parameters that define the geometry of the first 7 generations (asymmetric zone), to simulate the different tests for a specific patient. Of course, this requires detailed information on the geometry of the first generations of the patient's lung, but this is entirely possible with current imaging techniques [ref added].

Still about imaging, we know that conventional ventilation tests can give contradictory results, and that dynamic/functional imaging seems to be a more accurate alternative method for characterizing regional ventilation and gas concentration in the lungs [ref added]. At this stage, the code is not intended to offer a comparison with the results of such a technique, but this could be an interesting prospect for further development.

Minor comments:

1. Line 77 – Please use the unabbreviated version of GOLD (Global Initiative for Chronic Obstructive Lung Disease) when first mentioning it.

OK, done.

2. Line 177 – The paragraph starting at line 177 is not clear. Please rewrite for improved clarity.

OK, we have reformulated it. It was indeed not very clear.

3. Figure 4 – Please alter the title of Fig. 4 to provide more details, e.g., "Probability of alveolar occurrence as a function of airway generation". (The mass used can be moved to within the plot or to the captions).

OK, done, thanks for the suggestion.

4. Figure 6 – Please add the standard deviation caused by the variation in airway resistance to the curve. (It can be done at select points).

We have preferred to indicate the 95% confidence interval, reflecting the fact that, for a given boy mass, there is a natural variation in lung geometry, and that the same percentage of constriction does not always correspond to the same choice of airways.

5. Line 367, 374 – Please fix the "Error! Reference source not found." in these lines.

Done, sorry we didn't see this when submitting the article.

6. Please revise Figure 8 to improve the readability of the curves. The light blue is very difficult to follow.

Done. We hope that it is OK now.
