# Supplementary material for: A new computational framework for simulating airway resistance, fraction of exhaled nitric oxide, and diffusing capacity for nitric oxide
Source: PLoS One. 2025 Jan 30;20(1):e0311667. doi: 10.1371/journal.pone.0311667 (PMC11781630; doi:10.1371/journal.pone.0311667)
Supplement: Peer review history

# Peer review: Decision Letter 0

**DOI:** 10.1371/journal.pone.0311667.r001

**Authors:** James West

7 Aug 2024

PONE-D-24-20339A new computational framework for simulating airway resistance, fraction of exhaled nitric oxide, and diffusing capacity for nitric oxidePLOS ONE

Dear Dr. Haut,

Thank you for submitting your manuscript to PLOS ONE. After careful consideration, we feel that it has merit but does not fully meet PLOS ONE’s publication criteria as it currently stands. Therefore, we invite you to submit a revised version of the manuscript that addresses the points raised during the review process.

Please submit your revised manuscript by Sep 21 2024 11:59PM. If you will need more time than this to complete your revisions, please reply to this message or contact the journal office at plosone@plos.org. When you're ready to submit your revision, log on to https://www.editorialmanager.com/pone/ and select the 'Submissions Needing Revision' folder to locate your manuscript file.

Please include the following items when submitting your revised manuscript:

- A rebuttal letter that responds to each point raised by the academic editor and reviewer(s). You should upload this letter as a separate file labeled 'Response to Reviewers'.
- A marked-up copy of your manuscript that highlights changes made to the original version. You should upload this as a separate file labeled 'Revised Manuscript with Track Changes'.
- An unmarked version of your revised paper without tracked changes. You should upload this as a separate file labeled 'Manuscript'.

If you would like to make changes to your financial disclosure, please include your updated statement in your cover letter. Guidelines for resubmitting your figure files are available below the reviewer comments at the end of this letter.

If applicable, we recommend that you deposit your laboratory protocols in protocols.io to enhance the reproducibility of your results. Protocols.io assigns your protocol its own identifier (DOI) so that it can be cited independently in the future. For instructions see: https://journals.plos.org/plosone/s/submission-guidelines#loc-laboratory-protocols. Additionally, PLOS ONE offers an option for publishing peer-reviewed Lab Protocol articles, which describe protocols hosted on protocols.io. Read more information on sharing protocols at https://plos.org/protocols?utm_medium=editorial-email&utm_source=authorletters&utm_campaign=protocols.

We look forward to receiving your revised manuscript.

Kind regards,

James West, PhD

Academic Editor

PLOS ONE

Journal requirements:   

When submitting your revision, we need you to address these additional requirements.

1. Please ensure that your manuscript meets PLOS ONE's style requirements, including those for file naming. The PLOS ONE style templates can be found at

https://journals.plos.org/plosone/s/file?id=wjVg/PLOSOne_formatting_sample_main_body.pdf and

https://journals.plos.org/plosone/s/file?id=ba62/PLOSOne_formatting_sample_title_authors_affiliations.pdf

2. We note that Figures 1 and 2 in your submission contain [map/satellite] images which may be copyrighted. All PLOS content is published under the Creative Commons Attribution License (CC BY 4.0), which means that the manuscript, images, and Supporting Information files will be freely available online, and any third party is permitted to access, download, copy, distribute, and use these materials in any way, even commercially, with proper attribution. For these reasons, we cannot publish previously copyrighted maps or satellite images created using proprietary data, such as Google software (Google Maps, Street View, and Earth). For more information, see our copyright guidelines: http://journals.plos.org/plosone/s/licenses-and-copyright.

We require you to either (1) present written permission from the copyright holder to publish these figures specifically under the CC BY 4.0 license, or (2) remove the figures from your submission:

1. You may seek permission from the original copyright holder of Figures 1 and 2 to publish the content specifically under the CC BY 4.0 license.  

We recommend that you contact the original copyright holder with the Content Permission Form (http://journals.plos.org/plosone/s/file?id=7c09/content-permission-form.pdf) and the following text:

“I request permission for the open-access journal PLOS ONE to publish XXX under the Creative Commons Attribution License (CCAL) CC BY 4.0 (http://creativecommons.org/licenses/by/4.0/). Please be aware that this license allows unrestricted use and distribution, even commercially, by third parties. Please reply and provide explicit written permission to publish XXX under a CC BY license and complete the attached form.”

Please upload the completed Content Permission Form or other proof of granted permissions as an ""Other"" file with your submission.

In the figure caption of the copyrighted figure, please include the following text: “Reprinted from [ref] under a CC BY license, with permission from [name of publisher], original copyright [original copyright year].”

2. If you are unable to obtain permission from the original copyright holder to publish these figures under the CC BY 4.0 license or if the copyright holder’s requirements are incompatible with the CC BY 4.0 license, please either i) remove the figure or ii) supply a replacement figure that complies with the CC BY 4.0 license. Please check copyright information on all replacement figures and update the figure caption with source information. If applicable, please specify in the figure caption text when a figure is similar but not identical to the original image and is therefore for illustrative purposes only.

The following resources for replacing copyrighted map figures may be helpful:

USGS National Map Viewer (public domain): http://viewer.nationalmap.gov/viewer/

The Gateway to Astronaut Photography of Earth (public domain): http://eol.jsc.nasa.gov/sseop/clickmap/

Maps at the CIA (public domain): https://www.cia.gov/library/publications/the-world-factbook/index.html and https://www.cia.gov/library/publications/cia-maps-publications/index.html

NASA Earth Observatory (public domain): http://earthobservatory.nasa.gov/

Landsat: http://landsat.visibleearth.nasa.gov/

USGS EROS (Earth Resources Observatory and Science (EROS) Center) (public domain): http://eros.usgs.gov/#

Natural Earth (public domain): http://www.naturalearthdata.com/

3. Please review your reference list to ensure that it is complete and correct. If you have cited papers that have been retracted, please include the rationale for doing so in the manuscript text, or remove these references and replace them with relevant current references. Any changes to the reference list should be mentioned in the rebuttal letter that accompanies your revised manuscript. If you need to cite a retracted article, indicate the article’s retracted status in the References list and also include a citation and full reference for the retraction notice.

Additional Editor Comments (if provided):

[Note: HTML markup is below. Please do not edit.]

Reviewers' comments:

Reviewer's Responses to Questions

**Comments to the Author**

1. Is the manuscript technically sound, and do the data support the conclusions?

The manuscript must describe a technically sound piece of scientific research with data that supports the conclusions. Experiments must have been conducted rigorously, with appropriate controls, replication, and sample sizes. The conclusions must be drawn appropriately based on the data presented. 

Reviewer #1: Yes

**********

2. Has the statistical analysis been performed appropriately and rigorously? 

Reviewer #1: Yes

**********

3. Have the authors made all data underlying the findings in their manuscript fully available?

The PLOS Data policy requires authors to make all data underlying the findings described in their manuscript fully available without restriction, with rare exception (please refer to the Data Availability Statement in the manuscript PDF file). The data should be provided as part of the manuscript or its supporting information, or deposited to a public repository. For example, in addition to summary statistics, the data points behind means, medians and variance measures should be available. If there are restrictions on publicly sharing data—e.g. participant privacy or use of data from a third party—those must be specified.

Reviewer #1: Yes

**********

4. Is the manuscript presented in an intelligible fashion and written in standard English?

PLOS ONE does not copyedit accepted manuscripts, so the language in submitted articles must be clear, correct, and unambiguous. Any typographical or grammatical errors should be corrected at revision, so please note any specific errors here.

Reviewer #1: Yes

**********

5. Review Comments to the Author

Please use the space provided to explain your answers to the questions above. You may also include additional comments for the author, including concerns about dual publication, research ethics, or publication ethics. (Please upload your review as an attachment if it exceeds 20,000 characters)

Reviewer #1: This manuscript presents a computational framework to estimate the airway resistance in the airway tree and to simulate the diffusing capacity for nitric oxide (DLNO) test used to assess lung function in diseases such as asthma and chronic obstructive pulmonary disease (COPD). The presented model can account for variation in lung geometry using body mass and allows for the simulation of heterogeneities.

However, several points are not explained well or remain unclear. These points have been listed below.

Major Comments:

1. Figures 4, 6, 7, 8, 10, and 11 all have used three different values of body mass. Please use a consistent value or report the curves for all the values.

2. Figure 8 – Could you please elaborate as to why the NO concentration is discontinuous at the branching points?

3. While the study results indicate that the model can capture the average behavior for a given body weight, could the authors discuss how the model could be used to simulate outliers in a subject-specific manner?

4. While the ventilation tests offer confounded results, dynamic/functional imaging appears to be a method to determine regional ventilation and gas concentration. Could the authors discuss the possibility of estimating regional ventilation/NO concentration that matches the observed scans?

a. In addition, medical imaging has emerged as a significant diagnostic tool in assessing lung health/function. The need to create patient-specific lung geometry and airways from medical images will be relevant to this study. Please add a brief discussion in the Introduction and Discussion and cite relevant works such as https://doi.org/10.1117/12.2653648 and https://doi.org/10.1111/j.1440-1843.2011.02117.x. Also, the synthetic generation of airways (https://doi.org/10.1117/12.3006973) is relevant to this study.

5. Please include a limitation subsection in section 3, discussing potential limitations and avenues for improvement.

Minor comments:

1. Line 77 – Please use the unabbreviated version of GOLD (Global Initiative for Chronic Obstructive Lung Disease) when first mentioning it.

2. Line 177 – The paragraph starting at line 177 is not clear. Please rewrite for improved clarity.

3. Figure 4 – Please alter the title of Fig. 4 to provide more details, e.g., “Probability of alveolar occurrence as a function of airway generation”. (The mass used can be moved to within the plot or to the captions).

4. Figure 6 – Please add the standard deviation caused by the variation in airway resistance to the curve. (It can be done at select points).

5. Line 367, 374 – Please fix the “Error! Reference source not found.” in these lines.

6. Please revise Figure 8 to improve the readability of the curves. The light blue is very difficult to follow.

**********

6. PLOS authors have the option to publish the peer review history of their article (what does this mean?). If published, this will include your full peer review and any attached files.

If you choose “no”, your identity will remain anonymous but your review may still be made public.

**Do you want your identity to be public for this peer review?** For information about this choice, including consent withdrawal, please see our Privacy Policy.

Reviewer #1: No

**********

[NOTE: If reviewer comments were submitted as an attachment file, they will be attached to this email and accessible via the submission site. Please log into your account, locate the manuscript record, and check for the action link "View Attachments". If this link does not appear, there are no attachment files.]

While revising your submission, please upload your figure files to the Preflight Analysis and Conversion Engine (PACE) digital diagnostic tool, https://pacev2.apexcovantage.com/. PACE helps ensure that figures meet PLOS requirements. To use PACE, you must first register as a user. Registration is free. Then, login and navigate to the UPLOAD tab, where you will find detailed instructions on how to use the tool. If you encounter any issues or have any questions when using PACE, please email PLOS at figures@plos.org. Please note that Supporting Information files do not need this step.

# Author comment: Author response to Decision Letter 0

**DOI:** 10.1371/journal.pone.0311667.r002

18 Sep 2024

Dear Editor, Dear Reviewer,

Here are our responses to the editorial team's comments and the reviewer's comments. We believe we have responded to all of them.

I remain at your disposal for any further information.

Best regards,

Benoit Haut

Answer to editorial team:

The left side of Figure 1 was drawn from scratch by one of the authors (Cyril Karamaoun). The right side is already licensed under the Creative Commons Attribution 2.5, and this is properly referenced in the figure legend: "Image by Patrick J. Lynch, medical illustrator / Wikimedia Commons. License: Creative Commons Attribution 2.5 License 2006. Bronchial anatomy detail of alveoli and pulmonary circulation". (see: https://commons.wikimedia.org/wiki/File:Bronchial_anatomy.jpg). This image can be used freely. 

Figure 2 was drawn entirely from scratch by Clément Rigaut. 

The document format should meet all requirements. 

Three references have been added to this revised version at the request of the reviewers. All other references are to previously published articles.

Answers to the reviewers:

Major comments:

1. Figures 4, 6, 7, 8, 10, and 11 all have used three different values of body mass. Please use a consistent value or report the curves for all the values.

We have followed your recommendation, choosing a mass of 75 kg for all these figures (average for adults in Belgium), except for figure 7. For this figure, we prefer to retain a mass of 80 kg (average for adult men only), as the results are compared with Silkoff's data, obtained mainly for men (8 out of 10 subjects). When a figure has been modified, the corresponding text has been adapted if necessary.

2. Figure 8 – Could you please elaborate as to why the NO concentration is discontinuous at the branching points?

Yes, of course. We have added the following comment in the text (page 17):

The discontinuities observed in the NO concentration profiles occur at the junctions between airways in the asymmetric zone. At this point, two airflows meet during exhalation, and these two flows have a priori different NO concentrations because they have different flow rates and have traveled a different path from the alveolar sacs (i.e., they have received different amounts of NO from the bronchial epithelium). Instantaneous mixing at the junction causes a jump in concentration, which becomes equal to the average of the concentrations in the two flows, weighted by their flow rates.

3. While the study results indicate that the model can capture the average behavior for a given body weight, could the authors discuss how the model could be used to simulate outliers in a subject-specific manner?

Currently, the user of the computational framework only needs to specify the subject's mass, and the geometry of the lungs is automatically generated by the code. Thus, a “standard” geometry is generated, which does not allow for the consideration of a patient that is an outlier in term of lung morphology. However, a simple extension of the code could be to allow the user to modify the parameters that define the geometry of the first 7 generations (asymmetric zone), to simulate the different tests for a specific patient.

We have added a comment about this in a discussion about future improvements (page 24). 

4. While the ventilation tests offer confounded results, dynamic/functional imaging appears to be a method to determine regional ventilation and gas concentration. Could the authors discuss the possibility of estimating regional ventilation/NO concentration that matches the observed scans? In addition, medical Imaging has emerged as a significant diagnostic tool In assessing lung health/function. The need to create patient-specific lung geometry and airways from medical images will be relevant to this study. Please add a brief discussion in the Introduction and Discussion and cite relevant works such as:

https://doi.org/10.1117/12.2653648

https://doi.org/10.1111/j.1440-1843.2011.02117.x. 

Also, the synthetic generation of airways (https://doi.org/10.1117/12.3006973) is relevant to this study.

Thanks for this valuable comment. We have added these three citations in the introduction / conclusion, thanks for the info. In particular, they are introduced in support of the discussion on future developments of the model (see below) and on the relevance of medical imaging as a diagnostic tool (introduction).

5. Please include a limitation subsection in section 3, discussing potential limitations and avenues for improvement.

Thanks for the advice. We have added the following discussion about some limitations and future improvements of the computational framework. However, instead of presenting it in Section 3, we think this discussion is more appropriate in the conclusion, since the limitations naturally call for future developments, which are well presented in a conclusion.

Currently, the user of the computational framework only needs to specify the subject's mass, and the geometry of the lung is automatically generated by the code. Thus, a "standard" geometry is generated, which does not allow for the consideration of a patient that is an outlier in term of lung morphology. However, a simple extension of the code could be to allow the user to modify the parameters that define the geometry of the first 7 generations (asymmetric zone), to simulate the different tests for a specific patient. Of course, this requires detailed information on the geometry of the first generations of the patient's lung, but this is entirely possible with current imaging techniques [ref added].

Still about imaging, we know that conventional ventilation tests can give contradictory results, and that dynamic/functional imaging seems to be a more accurate alternative method for characterizing regional ventilation and gas concentration in the lungs [ref added]. At this stage, the code is not intended to offer a comparison with the results of such a technique, but this could be an interesting prospect for further development. 

Minor comments:

1. Line 77 – Please use the unabbreviated version of GOLD (Global Initiative for Chronic Obstructive Lung Disease) when first mentioning it.

OK, done.

2. Line 177 – The paragraph starting at line 177 is not clear. Please rewrite for improved clarity.

OK, we have reformulated it. It was indeed not very clear.

3. Figure 4 – Please alter the title of Fig. 4 to provide more details, e.g., “Probability of alveolar occurrence as a function of airway generation”. (The mass used can be moved to within the plot or to the captions).

OK, done, thanks for the suggestion.

4. Figure 6 – Please add the standard deviation caused by the variation in airway resistance to the curve. (It can be done at select points).

We have preferred to indicate the 95% confidence interval, reflecting the fact that, for a given boy mass, there is a natural variation in lung geometry, and that the same percentage of constriction does not always correspond to the same choice of airways.

5. Line 367, 374 – Please fix the “Error! Reference source not found.” in these lines.

Done, sorry we didn't see this when submitting the article.

6. Please revise Figure 8 to improve the readability of the curves. The light blue is very difficult to follow.

Done. We hope that it is OK now.

Attachment Submitted filename: Answers to the reviewers.pdf

# Peer review: Decision Letter 1

**DOI:** 10.1371/journal.pone.0311667.r003

**Authors:** James West

24 Sep 2024

A new computational framework for simulating airway resistance, fraction of exhaled nitric oxide, and diffusing capacity for nitric oxide

PONE-D-24-20339R1

Dear Dr. Haut,

We’re pleased to inform you that your manuscript has been judged scientifically suitable for publication and will be formally accepted for publication once it meets all outstanding technical requirements.

Within one week, you’ll receive an e-mail detailing the required amendments. When these have been addressed, you’ll receive a formal acceptance letter and your manuscript will be scheduled for publication.

An invoice will be generated when your article is formally accepted. Please note, if your institution has a publishing partnership with PLOS and your article meets the relevant criteria, all or part of your publication costs will be covered. Please make sure your user information is up-to-date by logging into Editorial Manager at Editorial Manager® and clicking the ‘Update My Information' link at the top of the page. If you have any questions relating to publication charges, please contact our Author Billing department directly at authorbilling@plos.org.

If your institution or institutions have a press office, please notify them about your upcoming paper to help maximize its impact. If they’ll be preparing press materials, please inform our press team as soon as possible -- no later than 48 hours after receiving the formal acceptance. Your manuscript will remain under strict press embargo until 2 pm Eastern Time on the date of publication. For more information, please contact onepress@plos.org.

Kind regards,

James West, PhD

Academic Editor

PLOS ONE

Additional Editor Comments (optional):

Reviewers' comments:

Reviewer's Responses to Questions

**Comments to the Author**

1. If the authors have adequately addressed your comments raised in a previous round of review and you feel that this manuscript is now acceptable for publication, you may indicate that here to bypass the “Comments to the Author” section, enter your conflict of interest statement in the “Confidential to Editor” section, and submit your "Accept" recommendation.

Reviewer #1: All comments have been addressed

**********

2. Is the manuscript technically sound, and do the data support the conclusions?

The manuscript must describe a technically sound piece of scientific research with data that supports the conclusions. Experiments must have been conducted rigorously, with appropriate controls, replication, and sample sizes. The conclusions must be drawn appropriately based on the data presented. 

Reviewer #1: Yes

**********

3. Has the statistical analysis been performed appropriately and rigorously? 

Reviewer #1: Yes

**********

4. Have the authors made all data underlying the findings in their manuscript fully available?

The PLOS Data policy requires authors to make all data underlying the findings described in their manuscript fully available without restriction, with rare exception (please refer to the Data Availability Statement in the manuscript PDF file). The data should be provided as part of the manuscript or its supporting information, or deposited to a public repository. For example, in addition to summary statistics, the data points behind means, medians and variance measures should be available. If there are restrictions on publicly sharing data—e.g. participant privacy or use of data from a third party—those must be specified.

Reviewer #1: Yes

**********

5. Is the manuscript presented in an intelligible fashion and written in standard English?

PLOS ONE does not copyedit accepted manuscripts, so the language in submitted articles must be clear, correct, and unambiguous. Any typographical or grammatical errors should be corrected at revision, so please note any specific errors here.

Reviewer #1: Yes

**********

6. Review Comments to the Author

Please use the space provided to explain your answers to the questions above. You may also include additional comments for the author, including concerns about dual publication, research ethics, or publication ethics. (Please upload your review as an attachment if it exceeds 20,000 characters)

Reviewer #1: (No Response)

**********

7. PLOS authors have the option to publish the peer review history of their article (what does this mean?). If published, this will include your full peer review and any attached files.

If you choose “no”, your identity will remain anonymous but your review may still be made public.

**Do you want your identity to be public for this peer review?** For information about this choice, including consent withdrawal, please see our Privacy Policy.

Reviewer #1: No

**********

# Editor report: Acceptance letter

**DOI:** 10.1371/journal.pone.0311667.r004

**Authors:** James West

11 Oct 2024

PONE-D-24-20339R1 

PLOS ONE

Dear Dr. Haut, 

I'm pleased to inform you that your manuscript has been deemed suitable for publication in PLOS ONE. Congratulations! Your manuscript is now being handed over to our production team.

At this stage, our production department will prepare your paper for publication. This includes ensuring the following:

* All references, tables, and figures are properly cited

* All relevant supporting information is included in the manuscript submission,

* There are no issues that prevent the paper from being properly typeset

If revisions are needed, the production department will contact you directly to resolve them. If no revisions are needed, you will receive an email when the publication date has been set. At this time, we do not offer pre-publication proofs to authors during production of the accepted work. Please keep in mind that we are working through a large volume of accepted articles, so please give us a few weeks to review your paper and let you know the next and final steps. 

Lastly, if your institution or institutions have a press office, please let them know about your upcoming paper now to help maximize its impact. If they'll be preparing press materials, please inform our press team within the next 48 hours. Your manuscript will remain under strict press embargo until 2 pm Eastern Time on the date of publication. For more information, please contact onepress@plos.org.

If we can help with anything else, please email us at customercare@plos.org.

Thank you for submitting your work to PLOS ONE and supporting open access. 

Kind regards, 

PLOS ONE Editorial Office Staff

on behalf of

Dr. James West 

Academic Editor

PLOS ONE
